# An open vision-language model for diverse medical applications

**DOI:** 10.1038/s41591-026-04626-w

**Published:** 2026-10-06

**Authors:** Andrew Sellergren, Sahar Kazemzadeh, Fereshteh Mahvar, Atilla Kiraly, Madeleine Traverse, Timo Kohlberger, Chufan Gao, Ronnachai Jaroensri, Shawn Xu, Fayaz Jamil, Cían Hughes, Charles Lau, Justin Chen, Liron Yatziv, Tiffany Chen, Bram Sterling, Stefanie Anna Baby, Susanna Maria Baby, Jeremy Lai, Samuel Schmidgall, Lu Yang, Kejia Chen, Per Bjornsson, Shashir Reddy, Ryan Brush, Kenneth Philbrick, Bashir Sadjad, Mercy Asiedu, Ines Mezerreg, Howard Hu, Howard Yang, Richa Tiwari, Sunny Jansen, Preeti Singh, Yubin Kim, Daniel McDuff, Catherine Kozlowski, Rachelle Sico, Aishwarya Kamath, Johan Ferret, Shreya Pathak, Nino Vieillard, Ramona Merhej, Sarah Perrin, Tatiana Matejovicova, Alexandre Ramé, Morgane Riviere, Louis Rouillard, Thomas Mesnard, Geoffrey Cideron, Jean-bastien Grill, Sabela Ramos, Edouard Yvinec, Michelle Casbon, Elena Buchatskaya, Jean-Baptiste Alayrac, Dmitry Lepikhin, Vlad Feinberg, Sebastian Borgeaud, Alek Andreev, Cassidy Hardin, Robert Dadashi, Léonard Hussenot, Armand Joulin, Olivier Bachem, Clement Farabet, Joelle Barral, Tris Warkentin, Jonathon Shlens, David Fleet, Shekoofeh Azizi, Victor Cotruta, Omar Sanseviero, Gus Martins, Phoebe Kirk, Anand Rao, Yossi Matias, Katherine Chou, Avinatan Hassidim, Dale Webster, Shravya Shetty, Kavi Goel, Yun Liu, Can Kirmizibayrak, David F. Steiner, Rory Pilgrim, Daniel Golden, Lin Yang

**Affiliations:** 1 Google, Mountain View, CA USA; 2 Google via Advanced Clinical, Deerfield, IL USA; 3 Google via Buchanan Ingersoll and Rooney PC, Pittsburgh, PA USA

**Keywords:** Medical imaging, Medical research, Health care

## Abstract

Artificial intelligence has high potential for impact in healthcare applications, but its training and deployment are challenging due to diverse data, a complex spectrum of possible tasks and important privacy needs. High-performing foundation models that enable data-efficient fine-tuning for diverse downstream tasks can meaningfully accelerate development in this domain. We introduce MedGemma, a collection of medical vision-language foundation models based on Gemma 3. MedGemma demonstrates advanced medical understanding and reasoning across images and text and multiple medical imaging domains, exceeding the performance of similarly sized generative models while maintaining the general capabilities of the Gemma base models. For out-of-distribution tasks, MedGemma achieves improvements of 2.6–10% in medical image question answering, 15.5–18.1% in chest X-ray finding classification and 10.8% in agentic evaluations compared with the base models. Our results show that fine-tuning MedGemma can be more effective than fine-tuning the base Gemma 3 model for medical tasks, particularly in the setting of limited training data. We additionally introduce MedSigLIP, a medically tuned vision encoder derived from SigLIP. MedSigLIP powers the visual understanding capabilities of MedGemma and, as an encoder, achieves performance comparable to or better than that of many specialized medical image encoders. Taken together, the MedGemma collection provides a strong foundation of medical image and text capabilities, with the potential to accelerate medical research and the development of downstream applications.

## Abstract (web summary)

MedGemma, a collection of medical vision-language foundation models based on Gemma 3, demonstrates advanced medical understanding and reasoning across images and text and multiple medical imaging domains, exceeding the performance of similarly sized generative models while maintaining the general capabilities of the Gemma base models.

## Main

The landscape of modern healthcare is characterized by the generation and use of unprecedented volumes and diversity of data. Diagnosis, treatment and monitoring rely on connecting the dots between many different data sources and medical specialties. Recently developed large multimodal models—advanced artificial intelligence (AI) systems trained on massive and diverse datasets—exhibit remarkable capabilities in detecting complex patterns, generating coherent text and processing visual information^1–3^. These capabilities mark a potential paradigm shift in how AI can be used to assist with current healthcare workflows and extract new insights.

While general-purpose (nonmedically tuned) large multimodal models demonstrate impressively broad abilities, generic models can lack the nuanced medical understanding needed to interpret and reason about medical data in a robust way^4–6^. Recognizing this gap, we developed MedGemma, a collection of medically tuned, vision-language foundation models. Built upon the robust architecture of Gemma 3 (ref. ^7^), the MedGemma models are designed to interpret and reason about medical images and text while retaining the strong general-purpose capabilities of Gemma 3.

### MedGemma

**Fig. 1:**
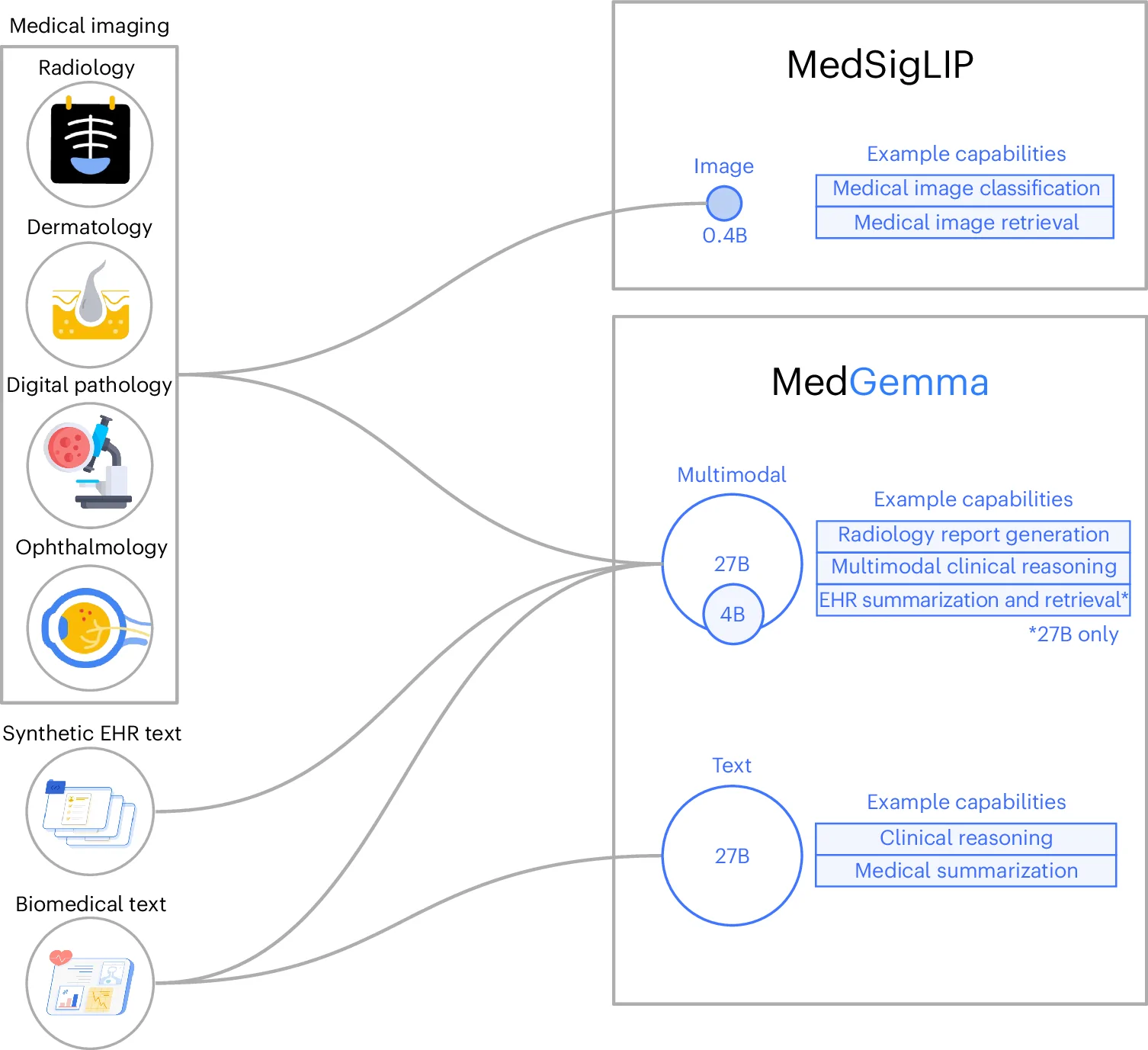
Overview of the MedGemma models. The MedGemma model collection features the MedSigLIP image encoder (400 million parameters), MedGemma 4B (4 billion parameters, multimodal), MedGemma 27B (27 billion parameters, multimodal) and MedGemma 27B Text (27 billion parameters, text only). Modalities used for domain-specific training of the respective models are indicated by the lines connecting data types on the left to models on the right. EHR, electronic health record.

We developed MedGemma as a collection of three model variants well suited for a variety of practical downstream applications (Fig. 1). Specifically, these include two multimodal variants, MedGemma 4B and MedGemma 27B (4 billion and 27 billion parameter models, respectively), which can accept text, images or both as input, and MedGemma 27B Text, which is optimized for text inputs. The multimodal MedGemma variants demonstrate strong performance on vision question answering (VQA) benchmarks, achieving performance similar to or exceeding prior models such as Med-Gemini^8,9^ and larger frontier models such as Gemini 2.5, despite being considerably smaller. MedGemma also demonstrates notable size-to-performance efficiency across benchmark tasks, including MedQA^10^, MedMCQA^11^, PubMedQA^12^, MMLU Med^13^, AfriMed-QA^14^ and AgentClinic^15^, outperforming open models of similar scale in our analysis.

The combination of strong benchmark results across both text and medical imaging tasks illustrates this high potential for MedGemma to be efficiently tuned and adapted to a diverse set of medical AI research and development efforts using a single base model. Additionally, MedGemma was optimized to retain the general-purpose language capabilities of Gemma 3, further enabling a particularly flexible and robust starting point for development.

MedGemma incorporates a specialized 400-million-parameter medical image encoder, MedSigLIP, specifically trained for medical imaging. To improve the encoder’s capabilities for medical imaging, we fine-tuned the vision encoder in Gemma 3 (SigLIP-400M)^7^ using millions of medical image–text pairs. These training data are described in Extended Data Table 1. MedSigLIP is the same encoder that powers MedGemma’s image interpretation capabilities. When used on its own, MedSigLIP enables data-efficient and zero-shot image classification and retrieval, with performance comparable to or exceeding specialized image encoders (summarized in Supplementary Note—MedSigLIP results).

Notably, these results represent only baseline capabilities, and the open nature of these models is intended to enable researchers worldwide to continue improving them for specific needs. In this regard, we also provide illustrative examples of fine-tuning for chest X-rays (CXRs), histopathology, dermatology and electronic health record question answering.

## Results

### MedGemma performance across medical text and imaging benchmarks

MedGemma performance was evaluated across several medical text and medical imaging benchmarks (Table 1), including both in-distribution benchmarks, for which a subset of the data was used for training or tuning with a held-out set for evaluation, and out-of-distribution (OOD) benchmarks, for which no subset of the data was used for training or tuning (note that, for general-purpose benchmarks, it is difficult to determine whether data are OOD given the large amount of pretraining data in the base model training mixture). Overall, MedGemma achieved accuracy exceeding or on par with much larger, nonopen models as well as task-specialized models across several benchmark tasks. The sections below outline performance across different categories, including medical text question answering, medical image classification, open-ended clinical reasoning, medical visual question answering, report generation and agentic behavior. The relevant model variants are included in the result tables below, with the complete set of performance results across MedGemma variants provided in Supplementary Note—Additional training data details.

**Table 1:** Overview of MedGemma evaluation datasets

Task	Dataset	Modality	No. of examples	OOD^a^
Medical text question answering	MedQA	Text	1,273	–
	MedMCQA	Text	4,183	–
	PubMedQA	Text	500	–
	MMLU Med	Text	3,685	–
	MedXpertQA	Text	2,450	*✓*
	AfriMed-QA (MCQ)	Text	25	–
	EHRQA	Text	4,377	–
	MultiMedQA (subset; open ended)	Text	130	*✓*
Medical image classification	MIMIC-CXR (Med-Gemini test set)	Radiology	1,532	–
	MIMIC-CXR (MAIRA test set)	Radiology	2,461	–
	ChestX-ray14 (CXR14)	Radiology	1,962	*✓*
	CheXpert	Radiology	668	*✓*
	US-Derm MCQA	Dermatology	1,996	–
	PathMCQA	Histopathology	450	–
	EyePACS	Ophthalmology	3,161	–
Medical visual question answering	MedXpertQA	General medical	2,000	*✓*
	SLAKE (English only)	Radiology	1,061	–
	VQA-RAD	Radiology	2,248	–
CXR report generation	MIMIC-CXR	Radiology	306	–
Medical agentic behavior	AgentClinic-MedQA	Agentic text	215	–
	AgentClinic-MIMIC-IV	Agentic text	200	*✓*
General purpose	MMLU Pro	Text	12,032	N/A
	Global MMLU Lite	Text	6,400	N/A
	MMMU (val)	Text + image	900	N/A

^a^OOD indicates data sources not seen during any model development stages.

N/A, not applicable.

#### Medical text question answering

Across all text-only biomedical question-answering tasks evaluated, MedGemma demonstrated superior performance to the ‘baseline’ Gemma 3 model, as well as competitive performance with much larger models in many cases (Table 2). Confidence intervals for the results, where calculated, are shown in Supplementary Table 1. Acknowledging that frontier models often still outperform smaller open models, these results highlight the clear benefit of domain specialization and position MedGemma as a useful starting point for many downstream applications. This evaluation was based on MedQA, MedMCQA, PubMedQA, MMLU subsets, AfriMed-QA and the OOD MedXpertQA. Notably, MedGemma’s performance of 86.2 on MedQA represented the best performance for an open model at the time of its release, other than the 671-billion-parameter DeepSeek-R1 model (91.0)^16^, and was substantially higher than the next closest well-documented open model, OpenBioLLM 70B (78.2)^17^.

**Table 2:** Accuracy on medical text benchmarks

Model^a^	Open^b^	MedQA	MedMCQA	PubMedQA	MMLU anatomy	MMLU clinical knowledge	MMLU college biology	MMLU college medicine	MMLU medical genetics	MMLU professional medicine	MMLU virology	AfriMed-QA	MedXpertQA text only (OOD)	MedXpertQA multimodal (OOD)
Small models														
MedGemma 4B	*✓*	64.1	55.7	73.4	59.3	71.3	70.8	65.3	83.0	76.8	53.0	52.0	14.2	24.4
Gemma 3 4B	*✓*	52.4	45.7	68.4	54.1	70.2	77.8	63.6	74.0	65.4	42.8	48.0	11.6	22.3
MedGemma 27B Text^c^ (with test-time scaling^39^)	*✓*	**86.2**	**72.4**	76.2	**83.7**	86.0	**96.5**	**86.1**	**97.0**	**93.4**	**53.6**	**84.0**	**25.7**	**33.0**^c^
Gemma 3 27B	*✓*	74.9	62.6	73.4	74.8	86.0	93.8	78.6	91.0	85.7	51.2	68.0	15.7	29.8
BioMistral DARE 7B^6,d^	*✓*	51.1	48.7	77.7	55.8	62.3	66.9	58.0	67.0	61.4	N/A	N/A	–	–
JSL-MedLlama 3 8B v2.0 (ref. ^40^)^d^	*✓* ^e^	61.4^f^	61.2	74.2	71.9	78.1	82.6	71.1	83.0	78.7	N/A	N/A	–	–
OpenBioLLM 8B^17^^,d^	*✓*	59.0	56.9	74.1	69.8	76.1	84.2	68.0	86.1	78.2	N/A	N/A	–	–
IQVIA Med-R1 8B^41^^,d^	✗	73.3	63.3	76.4	72.6	78.5	88.2	72.8	87.0	84.9	N/A	N/A	–	–
Large models														
OpenBioLLM 70B^17^^,d^	*✓*	78.2	74.0	79.0	83.9	92.9	93.8	85.7	93.2	93.8	N/A	N/A	–	–
DeepSeek-R1 (ref. ^16^)	*✓*	91.0	78.8	77.2	91.1	91.7	98.6	**90.8**	99.0	95.6	56.0	**92.0**	–	–
Gemini 2.5 Flash	✗	91.5	79.7	76.2	91.1	91.3	96.5	85.0	100.0	97.1	56.0	84.0	36.2	47.4
Gemini 2.5 Pro	✗	**93.9**	83.6	75.8	**91.1**	91.7	**98.6**	**89.0**	**96.0**	**96.3**	56.0	84.0	43.1	58.9
GPT 4o	✗	91.0	76.0	78.4	87.4	89.4	94.4	87.9	98.0	94.1	**58.4**	80.0	–	–
GPT o3	✗	93.3	**83.9**	**80.8**	**92.6**	**94.7**	**99.3**	**90.2**	**100.0**	96.7	56.0	84.0	**54.6**	**67.5**

Metrics for MedQA correspond to the original four-option test set, unless otherwise specified. Metrics for external small models as well as OpenBioLLM 70B were obtained from prior reports for these models. Other metrics were computed internally as described in the evaluation section. The MedXpertQA multimodal subset is the only benchmark that is not exclusively text and is included for completeness. Bold values indicate the best results in each section. A dash (–) indicates ‘not calculated’.

^a^See Supplementary Note—General evaluation approach for inclusion criteria.

^b^Open-weight models.

^c^MedGemma 27B Text performance is reported for all benchmarks except the MedXpertQA multimodal subset, on which MedGemma 27B performance is reported.

^d^Prior reported results.

^e^Research use only.

^f^Results for the all-options version of the dataset.

The open nature of MedGemma has also enabled additional third-party evaluations. For medical text question answering, the analysis provided in ref. ^18^ further demonstrates the performance advantages conferred by MedGemma’s medical domain training relative to Gemma 3. One notable difference was the improved performance of MedGemma relative to Gemma 3 on the MedHallu benchmark evaluation^19^, which asks a model to distinguish between a correct answer and a feasible sounding, but incorrect answer as one way to better understand model reliability. On the established ‘hard set’ of the MedHallu task, MedGemma 4B scored 0.50 and Gemma 3 4B scored 0.09 (normalized performance score).

#### Medical image classification

Medical image interpretation is a key contribution of the multimodal MedGemma models. To provide performance insights across a diverse set of medical imaging domains, we used image-based classification tasks in radiology, histopathology, dermatology and ophthalmology. These domains were selected because they are most consistent with training data and intended capabilities of MedGemma. (Of note, addressing classification as a zero-shot generative task may not provide maximum performance as compared to an embedding-based, task-specific classifier, but these evaluations are intended to provide insights into the quality and performance of the underlying image encoder and the overall model capabilities.) Results are summarized in Table 3 for CXR, histopathology, dermatology, and retina image classification. Notably, MedGemma not only demonstrated substantially superior performance relative to the standard Gemma 3 baselines but also outperformed much larger API-based models.

**Table 3:** Accuracy on zero-shot medical image classification

Modality	Dataset	Metric	Small models				Large models		SoTA VLM^a^
			MedGemma 4B	Gemma 3 4B	MedGemma 27B	Gemma 3 27B	Gemini 2.5 Flash	Gemini 2.5 Pro	
CXR	MIMIC-CXR Med-Gemini test set^b^	Macro F1 (5)	88.9	81.2	**90.0**	71.7	81.0	85.8	90.7 Med-Gemini^9^
	MIMIC-CXR MAIRA test set	Macro F1 (5)	**40.5**	26.7	40.2	25.0	32.2	31.9	51.6 Med-PaLM M^42^
	CheXpert (OOD)	Macro F1 (5)	48.1	32.6	**49.9**	26.2	37.4	37.0	49.0^c^ RadVLM^43^
	CXR14 (OOD)^b^	Macro F1 (3)	**50.1**	32.0	45.3	31.4	36.6	39.2	46.7 Med-Gemini^9^
Histopathology	PathMCQA	Accuracy	69.8	37.1	**71.6**	42.2	46.9	42.7	–
Dermatology	US-Derm MCQA	Accuracy	**71.8**	52.5	71.7	66.9	78.4	81.0	–
Retina	EyePACS	Accuracy	64.9	14.4	**75.3**	20.3	17.5	27.7	–

Performance on CXR classification, formulated as a zero-shot generative task, and on histopathology, dermatology and retina image classification, formulated as zero-shot, multiple-choice tasks on internal datasets. For the MIMIC-CXR and CheXpert datasets, macro F1 is calculated for five conditions—atelectasis, cardiomegaly, consolidation, edema and pleural effusion. For CXR14, macro F1 is calculated for three conditions—lung opacity, pneumothorax and fracture. Bold values indicate the best results within the Small models section. Previously reported state-of-the-art (SoTA) results are also provided for comparison when available.

^a^State-of-the-art at the time of MedGemma model release in July 2025, based on prior reported results.

^b^Radiologist-adjudicated labels are used as described in ref. ^9^ and Supplementary Note—Evaluation parameters, so metrics may not be directly comparable to those reported in the literature for external models.

^c^RadVLM macro F1 was calculated based on the individual F1 scores reported and estimated from the associated bar chart.

#### Open-ended medical question answering and clinical reasoning

To further evaluate the quality of model output, generated responses to open-ended medical content questions were evaluated. Model responses to these questions were rated by three physicians per question (see Supplementary Note for additional details). This analysis demonstrated a relative decrease in factually inaccurate responses of approximately 17% for MedGemma 4B versus Gemma 3 4B (inaccurate ratings for 18.5% of Gemma 3 responses versus 15.4% of MedGemma responses; Supplementary Fig. 1). While the decrease in inaccurate responses for MedGemma suggests a benefit of the medically specialized model, this type of evaluation is subject to potential interrater variability and application-specific considerations, highlighting the importance of use-case-specific fine-tuning and evaluation.

MedGemma is also capable of engaging in open-ended clinical reasoning tasks, a relatively unique capability for a model of its size. We explored this capability using open-ended clinical vignette prompts and examined the MedGemma text output through clinical expert review. Overall, MedGemma effectively summarized key components of the scenarios and identified relevant management recommendations. Examples of these tasks, along with clinical commentary, are shown in Supplementary Note—Medical reasoning examples.

#### Medical visual question answering

**Fig. 2:**
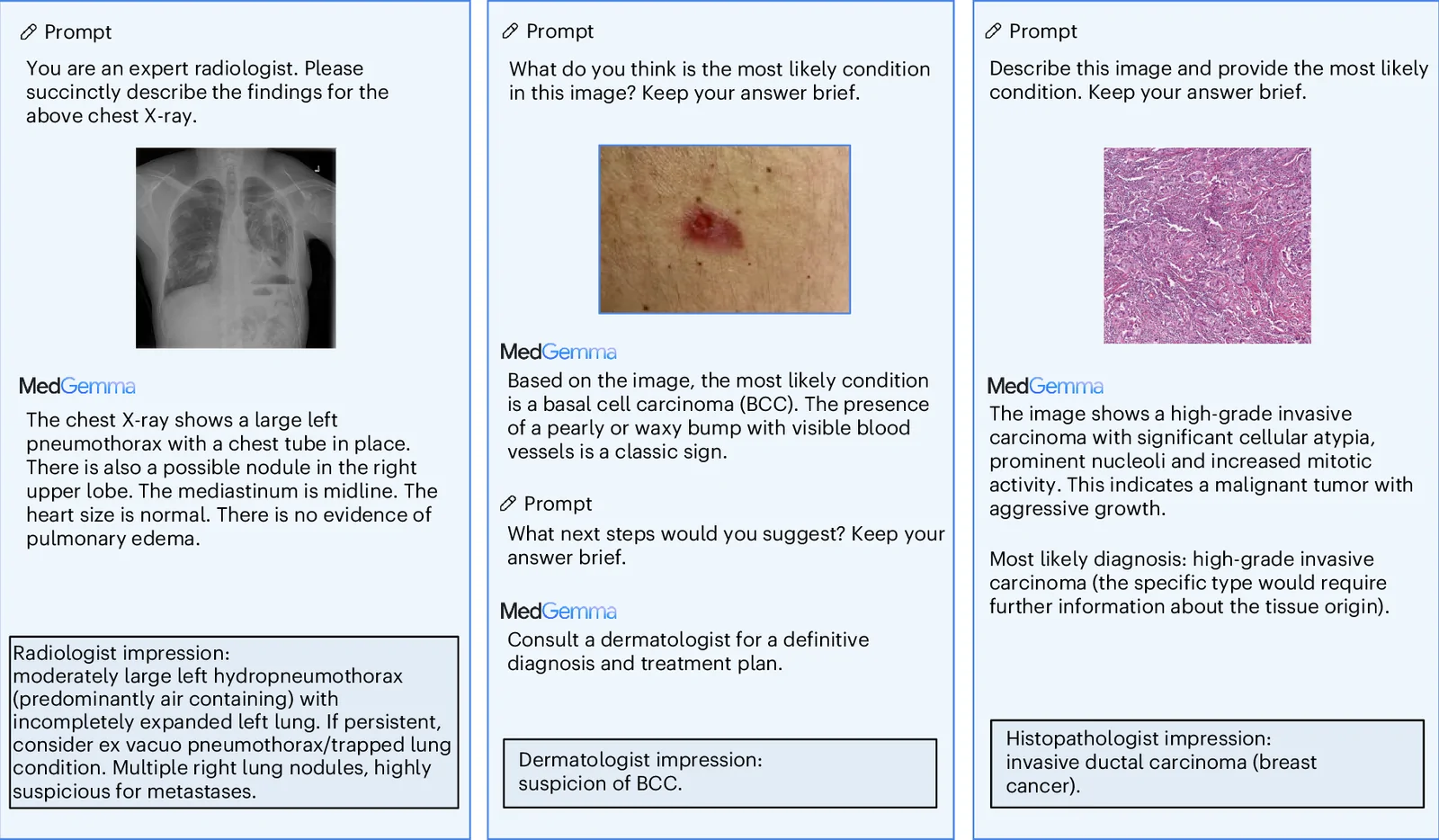
Examples of open-ended multimodal question answering with MedGemma 4B. Prompts that include both text and an image are shown along with the MedGemma 4B response. Assessments of the response by US board-certified clinicians are shown at the bottom of each section. X-ray image sourced from the NIH Clinical Center (https://nihcc.app.box.com/v/ChestXray-NIHCC/folder/37178474737), reproduced from ref. ^54^; dermatology image sourced from the Skin Condition Image Network dataset (https://github.com/google-research-datasets/scin), reproduced from ref. ^55^; and histopathology image sourced from The Cancer Genome Atlas^56^.

To evaluate medical VQA, we used the often-reported benchmarks SLAKE^20^ and VQA-RAD^21^. While different implementations for evaluating these benchmarks can make direct comparisons across literature challenging, we report direct comparisons of MedGemma to a number of additional models where possible as one component of demonstrating its strong baseline capabilities across modalities. Note that to avoid train–test contamination in VQA-RAD, we used the train and test splits described in refs. ^9,22^. These results are summarized in Extended Data Table 2. Again, MedGemma outperformed the standard Gemma 3 model and overall performed similarly to the much larger API-based models. LLaVA-Med and BiomedGPT-B are also included in the results table using the available reported metrics^23,24^, noting that these models were involved in supervised fine-tuning (SFT) for the specific tasks and datasets. In addition to these quantitative results, we show several illustrative examples of open-ended visual question answering in Fig. 2.

It is important to clarify that we did not compare MedGemma with specialized models in the literature for which these metrics were calculated using a discriminative, embedding-based approach. Such approaches can be informative but use the training set to define the set of possible text answers, and thus represent the task as a limited-vocabulary classification task rather than an open-ended VQA task. Similarly, while LLaVA-Tri^25^ also reports strong performance, the reported methodological details for these metrics are insufficient to support a direct comparison. Lastly, several other models have reported strong performance on these VQA benchmarks, such as HuatuoGPT-Vision^26^ and PMC-Cambrian-8B-AN^27^, with accuracy results reported in aggregate across open and closed questions. Given the challenge of comparing metrics even for the same benchmarks, we interpret the reported results for MedGemma on these VQA tasks as an important component towards demonstrating strong baseline performance across modalities.

#### CXR report generation

**Extended Data Fig. 1:**
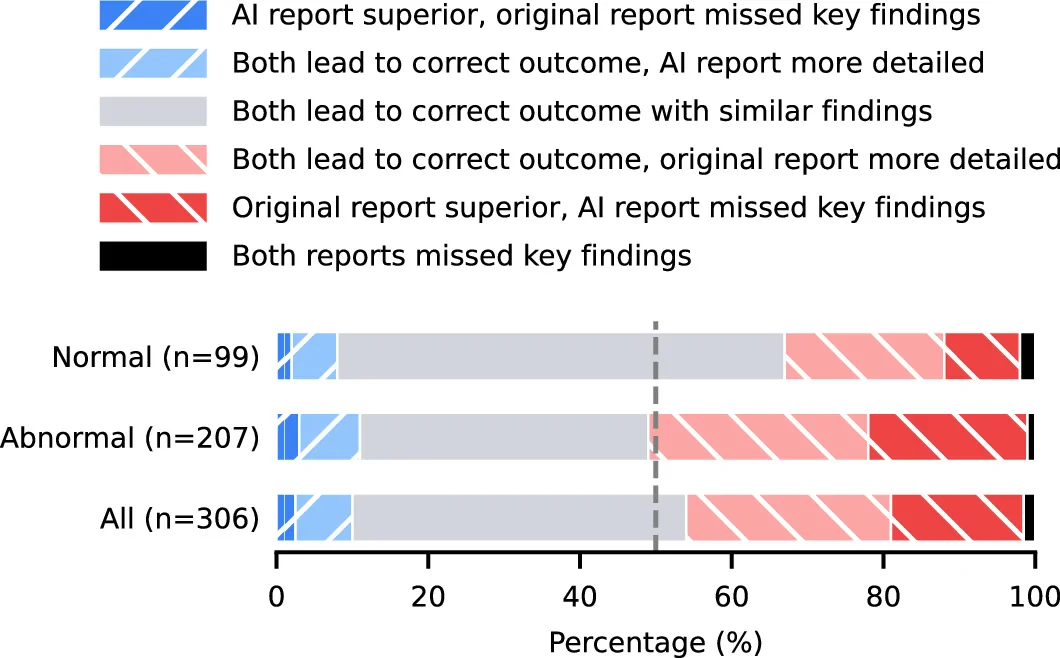
Human evaluation of chest X-ray report generation. The MedGemma 4B pretrained model was used to generate reports for a sample of images from the MIMIC-CXR test set, including images for which the original report identified abnormalities (‘abnormal’) and those for which the original report did not identify abnormalities (‘normal’). A single US board-certified thoracic radiologist reviewed the MIMIC-CXR report, the AI-generated report and the corresponding CXR image to judge the quality of the reports. Images were reviewed using the original DICOMs on a clinical diagnostic viewer. The radiologist was aware of which reports were AI generated. Across all cases, the radiologist rated 81% of MedGemma AI-generated CXR reports as likely to result in the same or superior clinical patient management when compared with the original reports.

Results of performance for CXR report generation are shown in Table 4. These results are based on the MIMIC-CXR dataset and the RadGraph F1 metric^28^. The pretrained checkpoint of MedGemma 4B demonstrates results similar to or better than other top-performing models, which is particularly notable given the comparator models are either larger or specialized only for this task. This shows the versatility of the Gemma architecture in adapting to complex medical use cases and enabling MedGemma. In addition, expert human evaluation demonstrated that 68% of generated reports from normal studies and 49% of generated reports from abnormal studies were rated as equal to or superior to the original reports (Extended Data Fig. 1). This corresponds to 81% of generated reports rated as likely to result in the same or superior clinical decisions as compared to the original reports. This represents superior performance to the larger Med-Gemini model, for which 73% of generated reports were rated as resulting in the same or superior clinical decisions as compared to the original reports^9^.

**Table 4:** Report generation on the MIMIC-CXR dataset

Model	Sections	Clinical metric (%)
		RadGraph F1
CXR-RePaiR^44^	F only	9.1
${M}^{2}$ transformer^45^	F only	22.0
Med-PaLM M, 12B^42^	F only	25.2
Med-PaLM M, 84B^42^	F only	26.7
MAIRA-1 (ref. ^46^)	F only	24.3
MAIRA-2 (ref. ^47^)	F only	**34.6**
R2Gen^48^	F + I	13.4
WCT^49^	F + I	14.3
CvT-21DistillGPT2 (ref. ^50^)	F + I	15.4
Flamingo-CXR^51^	F + I	20.5
Med-Gemini-2D^9^	F + I	24.4
PaliGemma 2 10B^52^	F + I	29.5
MedVersa^53^	F + I	30.0
MedGemma 4B PT	F + I	29.5
MedGemma 4B with RL	F + I	**30.3**

The ‘sections’ column indicates whether the model generates the findings (F) only or both the findings and impression (F + I) sections of the report; generation of findings and impressions is generally considered a more difficult problem as the impression section includes the radiologist’s comprehensive interpretation of the study. Previously reported RadGraph F1 metrics were sourced from existing published research except for MedGemma 4B PT and MedGemma 4B with task-specific RL, which were computed in this work. Higher scores are better. Bold values indicate the best results in each section. Please note that scores across models may not be directly comparable due to differences in inclusion and exclusion criteria across studies.

PT, pretrained.

Again, the open nature of MedGemma has enabled third-party evaluations. Specifically, for CXR report generation, the ReXrank leaderboard (ReXrank.ai) provides standardized evaluation across several models, datasets and metrics for CXR interpretation. Notably, MedGemma 4B demonstrates the highest Generative Radiology Report Evaluation and Error Notation (GREEN) score^29^ on the ReXGradient dataset^30^ (GREEN score 0.566) and the IU-Xray set^31^ (GREEN score 0.724), with middle-of-the-leaderboard performance on the MIMIC-III and CheXpert Plus datasets. MedGemma also demonstrates top-three performance on the ReXVQA medical VQA evaluation^32^. (ReXrank.ai, accessed 9 February 2026). While these results are consistent with strong baseline performance for MedGemma on CXR report generation and VQA, including on datasets not seen during training, the thorough ReXrank evaluation also notably illustrates that different models can excel on different metrics and datasets. As such, users can consider the aspects and metrics most important for their use case when selecting a model for further development and validation.

#### CXR anatomical structure localization

Although specific optimizations were not performed for this task, because anatomical bounding boxes were included in the training data for MedGemma 27B, we evaluated the localization of anatomical structures using the intersection over union (IoU) metric on the test set of the Chest ImaGenome dataset^33^. The findings are included in Supplementary Table 9 for reference, with IoU values of 0.16 for MedGemma 27B, 0.08 for baseline Gemma 3 27B, 0.03 for MedGemma 4B and 0.09 for Qwen3-VL 4B. These results demonstrate improved performance on this task when relevant training data are added, but indicate there is still substantial room for optimization and improvement of this capability.

#### Medical agentic behavior

To provide initial insights into the capabilities of MedGemma in the setting of use cases beyond single-turn question answering, we also evaluated MedGemma in an agentic role using the AgentClinic benchmark^15^. In this task, the model functions as a ‘physician agent’ in simulated clinical encounters, performing tasks such as patient history-taking, exam interpretation and diagnosis under uncertainty to reach the correct answers. Using the text-only AgentClinic-MedQA and AgentClinic-MIMIC-IV environments (with GPT 4o for the patient agent and measurement), MedGemma 27B exceeded the performance of human physicians on AgentClinic-MedQA and approached the performance of much larger models on both datasets. Results are summarized in Extended Data Table 3. Notably, we found that the smaller Gemma 3 4B and MedGemma 4B models were poorly suited to this task, demonstrating difficulty following system instructions within the AgentClinic agentic framework.

#### General purpose benchmarks

Given the limitations and poor performance that many medically specialized models exhibit when faced with nonmedical tasks, which may critically interweave with medical applications, we evaluated possible trade-offs of specialization. Models were evaluated on general language model benchmarks including MMLU Pro, Global MMLU Lite and MMMU datasets, with performance comparisons between MedGemma and Gemma 3 variants shown in Extended Data Table 4. MedGemma demonstrated only minor decreases in performance relative to the general models of the same size, further suggesting its potential utility for downstream applications that require both specialized and generalized capabilities, such as instruction following or diverse user interactions.

#### Observed themes

Several key themes emerged from our analysis. While larger, more computationally expensive models often performed better, medically specialized models such as MedGemma exhibited a distinct performance-efficiency advantage when their substantially smaller size was taken into account. This size-to-performance benefit was especially pronounced in medical vision-based tasks, where MedGemma surpassed the performance of substantially larger general-purpose models. Notably, there was a 500-fold difference in computational cost between MedGemma 4B and the most expensive comparator model, a crucial consideration for practical application development, where both development and compute usage are constraints.

We also observed that performance on older, established benchmarks tended to improve with newer models. While this observation reflects genuine advancements in model capabilities, it also raises the possibility of test data leakage, as these benchmarks are publicly available and frequently used for model development and evaluation.

### Fine-tuning MedGemma enables robust task-specific adaptation

While the MedGemma models achieved strong baseline performance, these models are intended as a useful starting point for specialized task adaptation (for example, specific styles, specific classification tasks, or other user needs).

**Extended Data Fig. 2:**
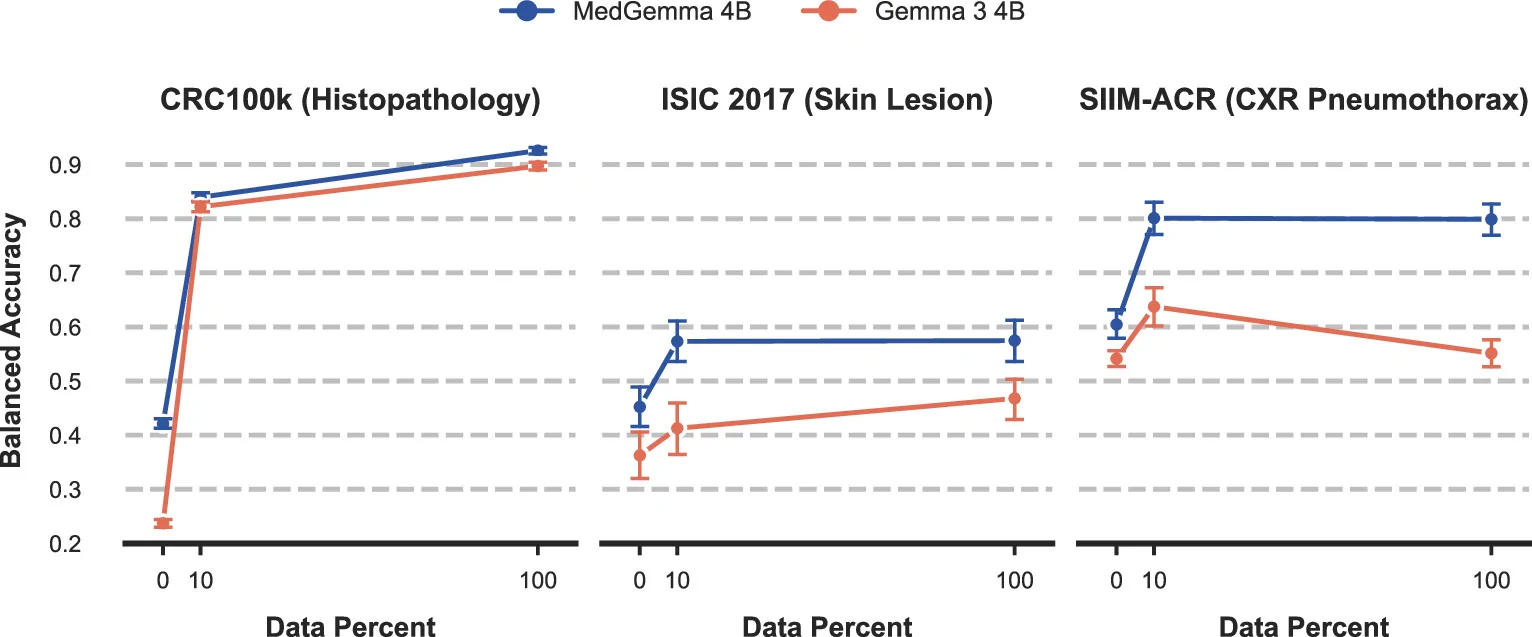
Fine-tuning performance of MedGemma 4B and Gemma 3 4B. Plots represent mean balanced accuracy ± 95% confidence intervals on independent test splits across varying training data fractions. To account for class imbalance, balanced accuracy was used and calculated as the unweighted mean of class-specific recall. A training fraction of 0% indicates the baseline model before fine-tuning. The unit of study was an individual image or tissue patch for histopathology. Sample sizes are: CRC100k (*n* = 7,180 patches), ISIC 2017 (*n* = 600 dermoscopic images) and SIIM ACR (*n* = 1,068 chest X-rays). Confidence intervals were derived via non-parametric bootstrap resampling with 1,000 iterations across independent test samples; no technical replicates or repeated measurements were used.

We conducted fine-tuning experiments using three different datasets (not previously included in MedGemma training) and using either MedGemma 4B or Gemma 3 4B as the base model. The results are shown in Extended Data Fig. 2 and generally demonstrate an increase in performance with fine-tuning and with higher performance for MedGemma 4B as compared to Gemma 3 4B. This performance advantage for MedGemma was particularly notable when using only 10% of the available data for a given task, supporting the notion of MedGemma as a useful starting point for data-efficient fine-tuning. We also note that for SIIM-ACR, Gemma 3 collapses to predicting the majority class, whereas MedGemma avoids this collapse, likely due, at least in part, to being pretrained on radiology data. While these results are provided to demonstrate illustrative comparisons of fine-tuning for classification tasks using a common SFT approach, specific fine-tuning methods and prompt engineering may benefit from further use-case-specific optimization.

Fine-tuning of MedGemma 4B with reinforcement learning (RL) was also performed to demonstrate further optimized performance for CXR report generation, as a task that was also included in MedGemma training. The resulting fine-tuned model achieved a RadGraph F1 score of 30.3 (for findings plus impression) on MIMIC-CXR, representing state-of-the-art performance for this specific metric and dataset, as shown in Table 4.

We also performed RL and evaluation experiments on EHRQA, a synthetic dataset specifically focused on longitudinal outpatient electronic health record data. Fine-tuning MedGemma 27B Text using RL on the EHRQA training set resulted in improved performance, with the greatest gains observed for question categories requiring reasoning across interdependent records. These results further support the potential of fine-tuning MedGemma for effective use in task-specific electronic health record applications. Additional details about the dataset and experiments are described in the Supplementary Note.

## Discussion

We introduce MedGemma, a new collection of medical vision-language foundation models, and MedSigLIP, a multidomain medical image encoder. These models were built on Gemma 3, with optimization for medical domains. We evaluated across a range of medical benchmarks that include tasks in clinical reasoning, biomedical knowledge, report generation and medical image classification, finding strong performance for MedGemma and MedSigLIP. Performance could be further improved through fine-tuning, highlighting the flexible nature of these models and their potential as useful starting points for developing AI applications in healthcare. The development of a vision encoder that can effectively tokenize across different medical image modalities combined with effective alignment for a performant language model represents key contributions of this work. As one concrete example of the utility of a single model that is capable across multiple modalities, developers have tuned MedGemma across different modalities using a unified pipeline, allowing more efficient and effective integration into a single system for general clinical practice guideline retrieval and navigation^34^.

With an increasing number of options available to developers building AI applications in healthcare, MedGemma provides specific advantages over general models. These advantages are largely due to the optimized incorporation of domain-specific data for both pretraining and post-training, as illustrated by improvements over base Gemma 3 models across all evaluated benchmarks and the achievement of performance on par with much larger models.

Compared with large or API-based frontier models, MedGemma offers advantages when the use case requires any of the following: a frozen model for documentation and reliability, sensitivity to training or inference costs, the ability to run locally or offline, specific medical image and text capabilities, or full control over model adaptation. The frontier models, on the other hand, may still be the preferred choice when the user requires optimal broad performance without the above constraints. Notably, large models may also be used in concert with models such as MedGemma in agentic settings to achieve useful combinations of capabilities.

The MedGemma collection of models enables a wide range of potential downstream applications for the developer community, with some examples described below. The multimodal capabilities, including access to image and text embeddings, may be particularly useful for medical image retrieval. This could aid interpretation by referencing similar past cases, as well as enable the development of research cohorts and the creation of educational tools. MedGemma enables the integration of diverse data, linking radiology, histopathology, ophthalmology and dermatology images with clinical information. The specialized text capabilities of the models can also extract key concepts from imaging reports and clinical notes, streamlining tasks such as matching patients for clinical trials, conducting pharmacovigilance reviews and analyzing healthcare quality metrics. The models’ ability to understand medical images and generate reports can also be fine-tuned to better assist radiologists and other clinicians in their workflow and improve how findings are communicated to patients. In addition to standalone use, these models can serve as powerful tools within agentic frameworks, combining capabilities across different modalities for customized and comprehensive solutions.

In this report, we evaluated the performance of MedGemma and MedSigLIP on a broad set of established benchmarks to provide a snapshot of their capabilities. However, we note that limitations exist for these benchmarks such that evaluating performance on them represents only the first step towards validating real-world utility^35,36^. Additionally, some benchmarks may be near saturation in terms of model performance, thus hindering the measurement of progress. As such, further work is warranted to continue evaluation of these models on new, high-quality (and more challenging) benchmarks aimed at better reflecting real-world utility, for example, in ref. ^37^. More work is also needed to understand the performance capabilities and requirements for actual application development, including their incorporation into agentic frameworks. These efforts will inform optimal use cases as well as development of future model versions with extended capabilities.

We also note that, across modalities, specialized models may still provide better performance or be better suited to some tasks. For example, while MedSigLIP and MedGemma demonstrate performance on par with many histopathology-specialized models for some tasks, state-of-the-art vision encoders for histopathology demonstrate superior performance on several benchmark tasks. Further optimization of pathology performance represents an area for possible improvement. Additional important areas for possible development include extending radiology capabilities to include three-dimensional series for CT and MRI, as well as capabilities for additional modalities such as genomics. Practitioners should compare MedGemma to other base models on their task of interest when possible. At the same time, while a specialized model might score higher on some tasks, a key strength of a multimodal medical foundation model such as MedGemma lies in its flexibility. Although MedGemma surpasses frontier model performance on some tasks (Table 3), its value also includes aspects other than performance, such as enabling data and model sovereignty, as well as computational efficiency. Instead of requiring separate adaptation and implementation approaches for each specific disease or image type, a unified approach can be applied to many tasks. This flexibility also extends to potential utility for use cases that benefit from language generation and multiple image modalities, enabling systems that can adapt to new medical challenges and help connect different aspects of patient information.

We openly released MedGemma and MedSigLIP to facilitate their widespread evaluation, improvement and adaptation by the community across multiple modalities. Initial examples from the broader research community of research performed on and with MedGemma following its initial release are summarized in Extended Data Table 5, including evaluations of OOD modalities and tasks. In addition to enabling evaluation and reproducibility, MedGemma’s openness provides developers with predictability and flexibility for extensive model adaptation and validation in real-world healthcare applications. We hope that this approach accelerates the development of AI applications across a broad array of healthcare use cases.

## Conclusion

In this study, we showed that MedGemma models demonstrate strong baseline performance across a variety of vision-language and text-only medical tasks, as well as the ability to further improve performance through task-specific fine-tuning. We also showed that MedSigLIP demonstrates robust multidomain capabilities and can thus serve as a strong medical vision encoder foundation model. These results demonstrate that optimizing approaches across pretraining and post-training can enable multimodal capabilities across several medical imaging domains, providing a unified starting point for further development. The breadth and efficiency of these models offer exciting possibilities to address a range of use cases. At the same time, the potential for model errors or subtle forms of distribution bias in training data remains an important aspect to address through continued model improvements, mitigation strategies and ongoing validation^38^. Thoughtful validation of safety, performance and reliability for downstream applications remains a critical aspect to advancing the use of multimodal AI models in medicine. By providing MedGemma and MedSigLIP models to the developer community with transparent methodologies, we hope to see them enable many useful and innovative medical applications.

## Methods

### Datasets

For general-purpose data replay during pretraining, the original data mixtures from SigLIP and Gemma 3 (ref. ^7^) were leveraged. The medical training and evaluation datasets largely followed those used in Med-Gemini^9^. Here we outline specific changes and differences in the datasets relative to the details described in ref. ^9^.

#### Training datasets

##### Text-only datasets

For text datasets, we sampled responses and logits from a large IT (instruction-tuned) teacher using the training splits of multiple medical question-answering datasets, including MedQA^10^, MedMCQA^11^, PubMedQA^12^, MedExpQA^57^, AfriMed-QA^14^, HealthSearchQA^58^, and LiveQA^59^. We also sampled responses and logits for approximately 200,000 synthetic medical questions. To generate these synthetic questions, we prompted Gemini with five randomly selected questions from the MedQA training split and asked for new questions based on, but nonoverlapping with, the provided examples. After generating an initial set of 500,000 questions, we reduced the final set to 200,000 with the goal of maintaining question diversity. This process involved vectorizing the questions (using TF-IDF with document frequency between 0.5 and 5), reducing dimensionality to 100 features, generating 100 clusters using *k*-means, and finally sampling an equally distributed set from these clusters.

##### Multimodal datasets

The multimodal capabilities of MedGemma are focused on two-dimensional medical images (for example, X-rays, two-dimensional slices from CT and MRI). Relative to Med-Gemini^9^, training on two-dimensional medical images used the same data sources, while the three-dimensional volumes and genomic datasets described in ref.^9^ were not included. Additionally, we and others have identified potential data quality issues in PathVQA and MedVQA. Thus, we removed them from the training dataset. We did not include PAD-UFES-20 in the post-training dataset because it focuses on six-class classification of very specific lesion types, which is not in line with the goal of more general-purpose dermatology capabilities and use cases. For the PMC-OA component of the training data, we included only single-panel medical images from PMC-OA for better data quality. Relative to Med-Gemini, we also introduced a larger internal collection for ophthalmology (184,852 more retina images), dermatology (51,049 more dermatology images with 210 different skin conditions), histopathology (a total of ~32.5 million patch–text pairs, downsampled during training) and radiology data (54,573 more CT 2D slices, 47,622 MRI 2D slices); see Extended Data Table 1. Additional details about the data sources comprising histopathology and radiology (CT-US1 and MRI-US1), along with the sampling and curation strategy, are described previously in ref. ^9^. CT and MRI slices used for training were curated based on text from the radiology reports specifying the location (that is, slice) of specific findings and representing the diversity of studies from a US-based radiology outpatient diagnostic center network. Histopathology patches were sampled across several datasets and tissue types using slide-level metadata and patch-level annotation to generate captions. Additional details on the datasets and captions are provided in Supplementary Tables 7 and 8. The internal dermatology dataset consists of de-identified dermatology images (both clinical and dermatoscopic) from Columbia, Australia and the US across teledermatology conditions, skin cancer, and normal skin, respectively. The Chest ImaGenome^33^ and EHRQA (Supplementary Note—Additional evaluation using an internal synthetic EHR dataset: EHRQA) datasets were used only for post-training of MedGemma 27B as additional areas of exploration for this model variant.

#### Data preprocessing

Our data preparation followed the study in ref.^9^ closely. Image padding and resizing algorithms remained the same, but because the vision encoder is different in Gemma 3, our images were resized to 896 × 896 instead of 768 × 768. Following Gemma 3, we used the SentencePiece tokenizer with 262,000 entries. Additionally, for CT images, we preselected the following three windows and converted them into the red, green and blue (RGB) color channels of the input image to highlight (1) bone and lung, window width of 2,250, window level of -100; (2) soft tissue, window width of 350, window level of 40; (3) brain, window width of 80, window level of 40.

### Modeling methodology

#### Modeling architecture and training infrastructure

The MedGemma model architecture follows Gemma 3 (ref. ^7^) and is compatible with all existing Gemma infrastructure. The Gemma 3 vision encoder is the 400M variant of the SigLIP encoder and is shared across the different Gemma language model sizes (4B and 27B). The input image resolution is 896 × 896 with pixel values normalized to (-1, 1). The language model component also follows Gemma 3, featuring arbitrary image–text interleaving and long context (128k). Similar to Gemma 3, MedGemma was trained on TPUv4, TPUv5e and TPUv5p, leveraged precomputed visual tokens for memory saving and used data and model sharding for multipod training.

#### Model training

The MedGemma multimodal models (MedGemma 4B and MedGemma 27B) used all of the following steps, while MedGemma 27B text leveraged the post-training stage alone (as we observed stronger text-only performance in initial tuning experiments with this approach).

##### Vision encoder enhancement for MedGemma

To improve the vision encoder’s ability to encode and distinguish subtle differences in medical images, we fine-tuned the vision encoder in Gemma 3 (SigLIP-400M) using over millions of medical image–text pairs as listed in Extended Data Table 1. To retain SigLIP’s existing performance, its original training data (for example, WebLI) were retained and medical data was mixed with a 2% weight during training.

To balance general-domain capabilities with specialized medical knowledge, the total proportion of medical data in the training mixture was constrained to 2%. Within this medical subset, data availability across modalities varied widely in scale. To fully use available data without inducing modality-specific overfitting, we used an iterative, empirical sampling strategy to define the final mixture weights. Specifically, sampling weights were optimized by tracking validation performance across a limited number of modality-specific tasks—CXR condition classification, pathology patch classification, diabetic retinopathy classification, dermatology condition classification and ImageNet zero-shot classification performance for general capability. Initial weights were set proportionally to the total number of examples per dataset, while limiting the total amount of medical data for training. After an initial training run, we analyzed the validation curves to adjust the distribution—for datasets on which the model overfit, weights were downsampled to match the number of examples seen at the step of peak performance. Conversely, for datasets on which validation metrics were still improving at the end of training, the sampling weights were doubled. This process was iterated until validation metrics across all modalities reached peak performance simultaneously toward the end of the training phase. The final optimized sampling distribution is detailed in Supplementary Table 5.

While the Gemma 3 vision encoder works at 896 × 896 resolution, we found that many medical vision tasks worked reasonably well at 448 × 448 resolution (Supplementary Table 2). Thus, while the MedGemma image encoder is based on 896 × 896 resolution for compatibility and consistency with Gemma 3, the released MedSigLIP model is based on 448 × 448 resolution for more efficient experimentation and adaptation by the community. The 448 × 448 encoder shares the same model weights as the 896 × 896 encoder, with the only difference being downsampled positional embeddings to work with fewer input patches at the lower resolution.

##### Multimodal decoder pretraining

After the vision encoder enhancement, the Gemma language model needed to be readapted to the new vision encoder, not only for medical data but also for the general image domain to preserve its visual-language reasoning capabilities. This goal was achieved in the pretraining stage by incorporating both the text and interleaved imaging data from the original mixture and the newly introduced medical domain image–text paired data. Notably, we did not introduce further medical text-only data in this step, as the original Gemma 3 mixture was already general-purpose and large-scale. To reduce compute requirements, we continued our pretraining on top of the original Gemma 3 pretrained checkpoints, mixed our medical image data (Extended Data Table 1) with 12% weight (based on tuning experiments), trained for approximately five epochs on the medical mixture given the mixing ratio, and selected the checkpoint based on the validation set performance on CXR report generation and radiology, dermatology and ophthalmology visual question answering.

For multimodal decoder training, the total proportion of medical data in the training mixture was constrained to approximately 12%. An empirical sampling strategy similar to that used for vision encoder enhancement was used to define the final mixture weights, with tuning performance across a limited number of modality-specific tasks: RadGraph F1 on MIMIC-CXR (radiology), ROUGE-L for patch captioning (pathology) and Top-1 classification accuracy for both dermatology (skin conditions) and ophthalmology (retinal imaging). As above, weights were adjusted based on peak performance or ongoing improvement at the end of the training run. The final optimized sampling distribution is detailed in Supplementary Table 6, ensuring that high-volume datasets were used effectively without dominating the training distribution or causing early overfitting.

##### Post-training

The knowledge acquired from pretraining needs to be surfaced as capabilities in the post-training stage. There are two primary post-training components as previously outlined for Gemma 3. The recipes for distillation and RL were the same as in Gemma 3 development, with the following additions: (1) Distillation—addition of medical text data during this component to enable further learning in these domains from a large instruction-tuned (IT) teacher. (2) RL—medical imaging data with paired text was used in the RL stage of post-training. For multimodal training, we found that RL enabled better generalization than SFT, so all multimodal post-training was performed via RL.

#### MedGemma fine-tuning

To demonstrate MedGemma’s capacity for improved task-specific performance relative to a nonmedical base model, we conducted fine-tuning experiments comparing use of MedGemma 4B and Gemma 3 4B as base models. These experiments included the following three multimodal tasks involving previously unseen datasets: (1) pneumothorax classification on CXRs via SIIM-ACR^60^, (2) histopathology patch classification via CRC100k^61^ and (3) skin lesion classification via ISIC 2017 (ref. ^62^). The base models were fine-tuned through SFT using low-rank adaptation using either all training data available (100%) or a 10% class-balanced selection to evaluate data efficiency. For SIIM-ACR and CRC100k, because these datasets provide no validation (tuning) splits, we reserved a random 10% per label of the original training data to form a validation set. For each task, we used a constant learning rate of 4 × 10^−5^ and fine-tuned the model up to four epochs on the full training dataset. Checkpoints that achieved the highest performance on the validation set were selected for the final evaluation on the test set. The balanced accuracy metric was selected to account for imbalanced label distribution in the test sets. Confidence intervals were generated by bootstrap resampling with 1,000 samples. Additionally, we performed RL-based fine-tuning for MIMIC-CXR report generation, starting with MedGemma 4B-IT as the base model, using the image and indication sections of the report as input, and training the model to complete the findings and impression sections.

#### Model evaluation

Model evaluation details for the different tasks are provided in Supplementary Note—MedGemma evaluations. Unless otherwise reported, all evaluations consisted of a single inference run per example. For MedGemma evaluations, a temperature of 0.0 was used for medical question sets and the default temperature was used on nonmedical benchmarks. For evaluations of all other models, on all datasets, each model’s default temperature and top-*k* settings were used. Additionally, details of the evaluation of the MedSigLIP encoder can be found in Supplementary Note—MedSigLIP evaluations. Evaluation using datasets completely external to training of the vision encoder, pretraining, or post-training is specified as OOD evaluation. All evaluations were conducted using data sources or data splits that were completely held out from MedGemma-specific training. Many members of the research community have also evaluated MedGemma and MedSigLIP in various scenarios following the release of the models^34,63–70^ and these results are summarized in Extended Data Table 5.

### Use of AI in paper preparation

The introduction section of this paper was drafted manually and then suggestions from Gemini were reviewed and incorporated where useful. Final manual checks were performed to ensure content accuracy. The authors take full responsibility for the content.

### Reporting summary

Further information on research design is available in the Nature Portfolio Reporting Summary linked to this article.

## Online content

Any methods, additional references, Nature Portfolio reporting summaries, source data, extended data, supplementary information, acknowledgements, peer review information; details of author contributions and competing interests; and statements of data and code availability are available at https://doi.org/10.1038/s41591-026-04626-w.

## Supplementary information

Supplementary Information Supplementary Note (description of the evaluation methodologies, datasets and text prompts used to assess the MedGemma and MedSigLIP models across a variety of medical and general-purpose tasks, as well as expanded training details, performance metrics, manual expert-review rubrics and clinical reasoning examples), Supplementary Figs. 1–5 and Supplementary Tables 1–25.

Reporting Summary

## Figures and Tables

**Extended Data Table 1:**
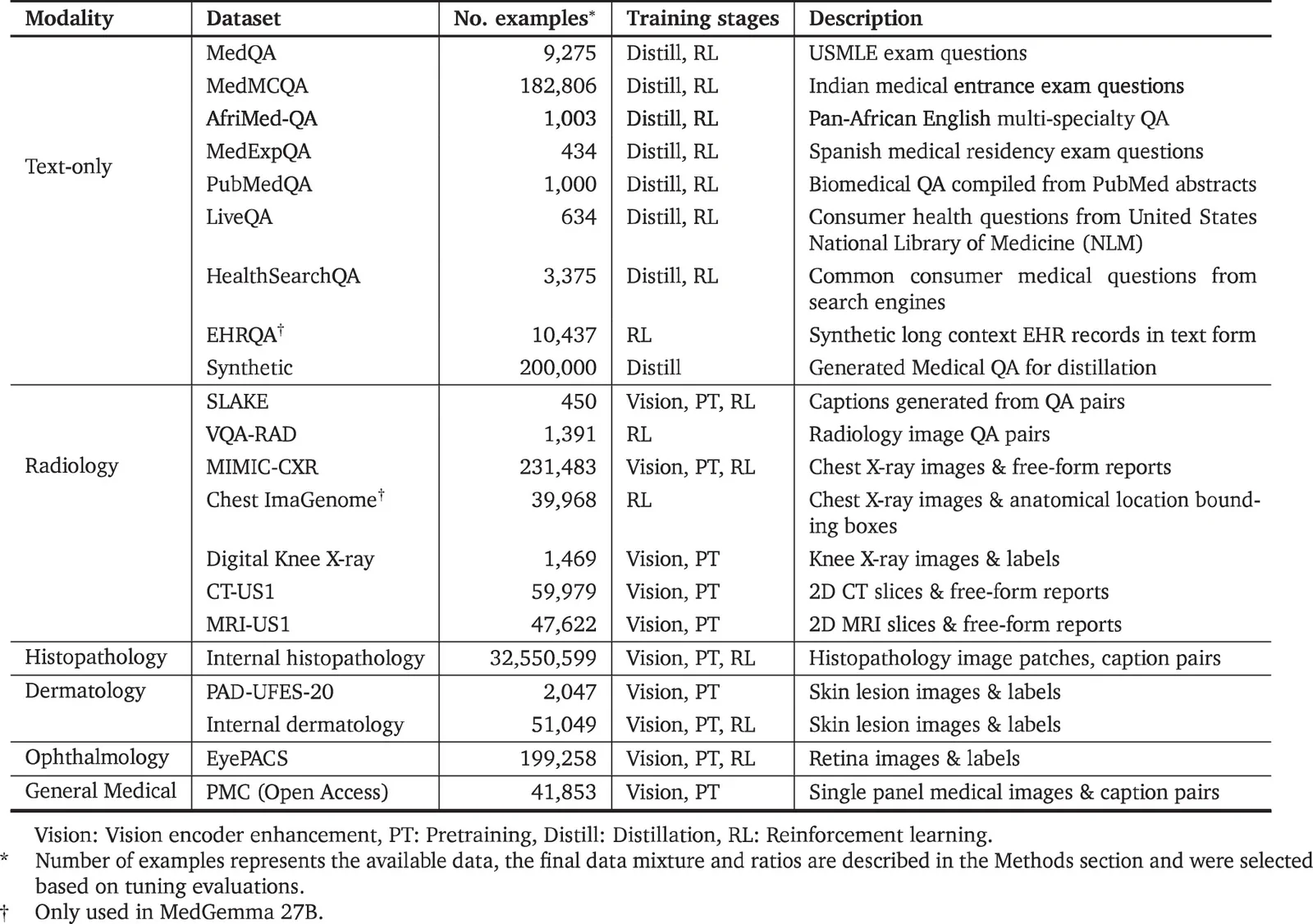
Overview of MedGemma training datasets

.

**Extended Data Table 2:**
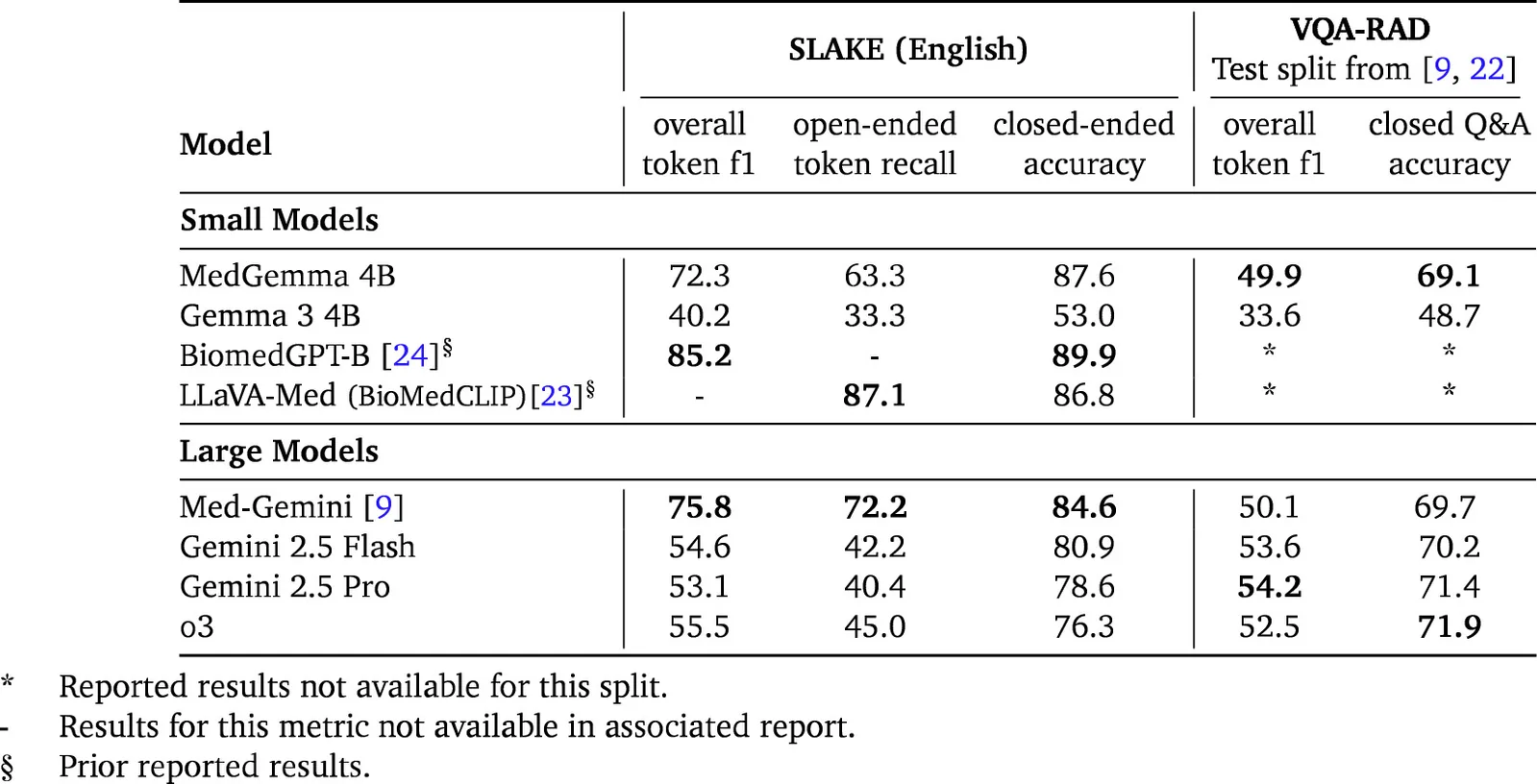
Medical VQA results for SLAKE and VQA-RAD

Bold values indicate the best results in each section. Note that the original VQA-RAD test set includes images that also appear in the train and test sets and we used the splits described in refs. ^9,22^ to avoid this contamination. The choice of these splits precludes direct comparison with externally reported results.

**Extended Data Table 3:**
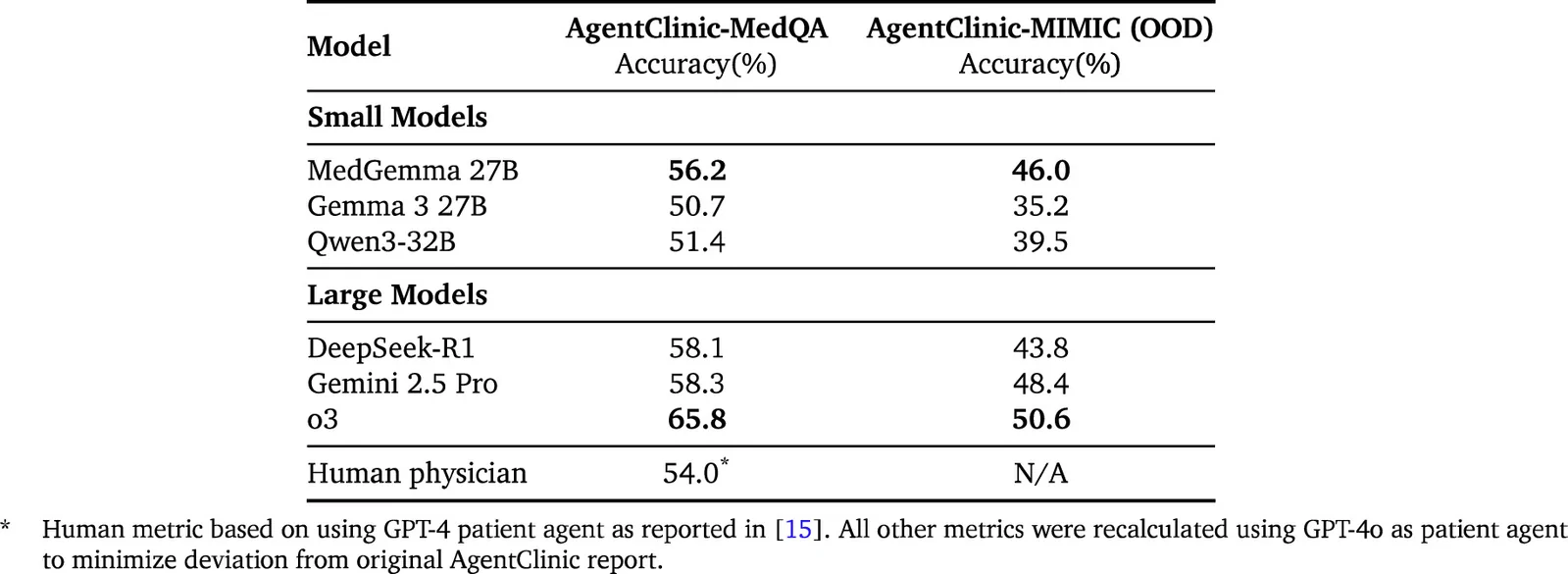
Performance on AgentClinic benchmarks

Bold values indicate the best results in each section. Performance of the 4B models is not reported as those models demonstrated difficulty following system instructions.

**Extended Data Table 4:**
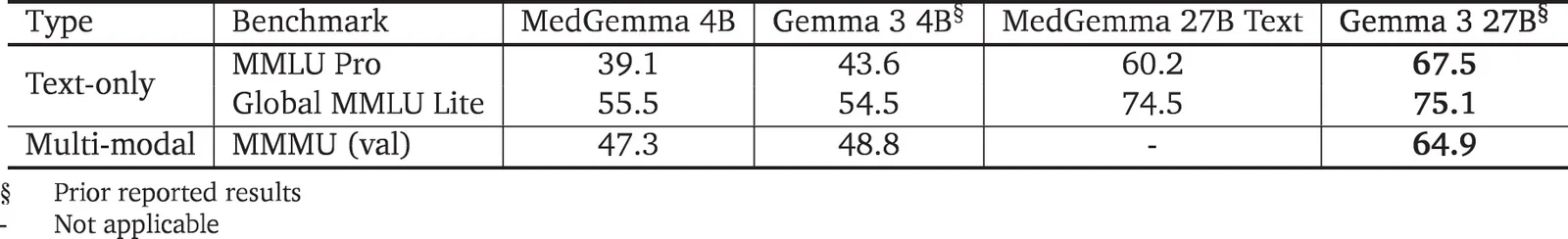
Accuracy results on general, non-medical benchmarks

Bold values indicate the best results in each section. Note that multimodal results are not applicable to MedGemma 27B Text.

**Extended Data Table 5:**
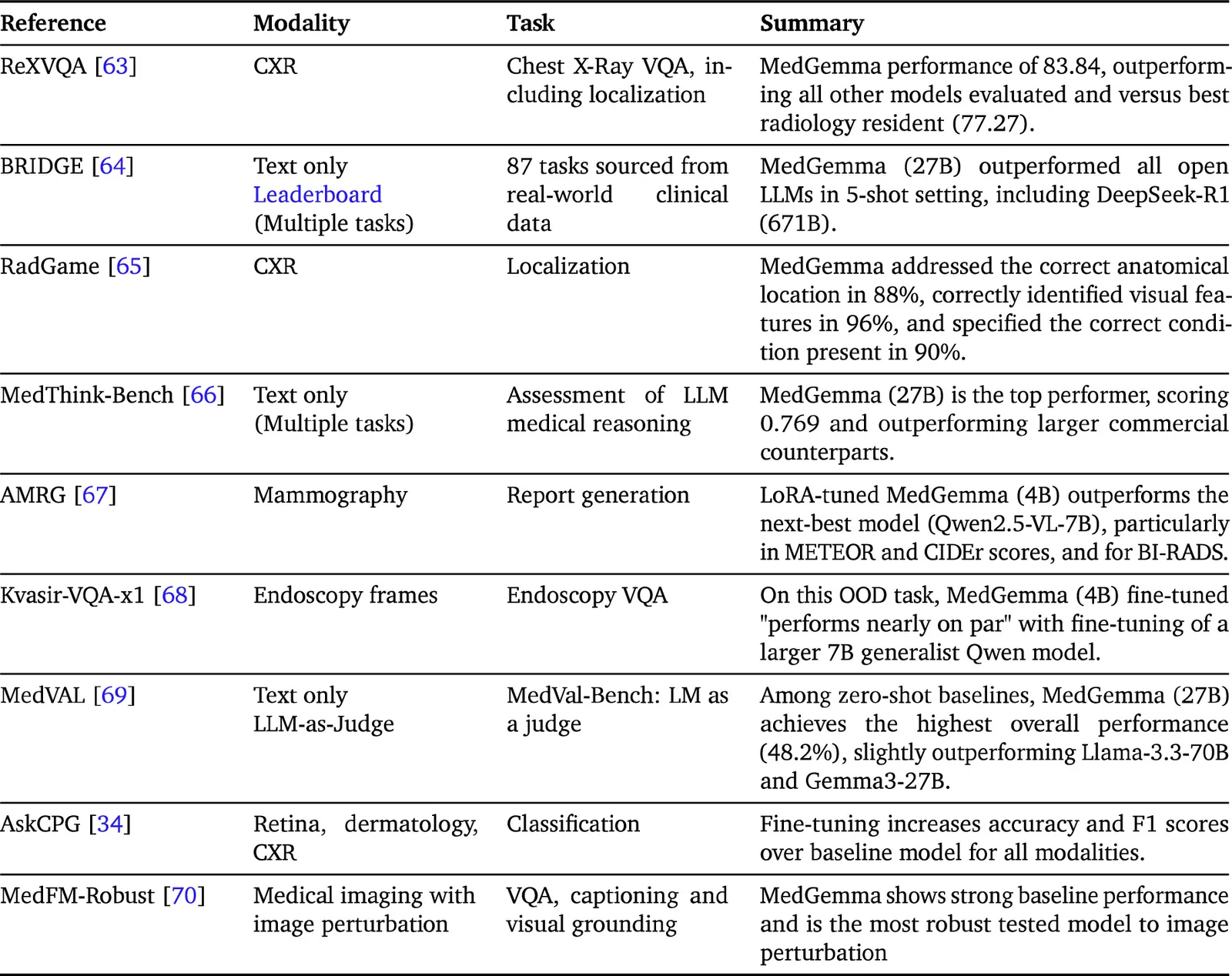
Examples from the broader research community of research performed on and with MedGemma since its initial release

## Data Availability

De-identified data from public and private datasets were used. Detailed datasets are listed in Table 1 and Extended Data Table 1. Due to licensing restrictions, we could not share the private datasets, including EHRQA, US-Derm MCQA, PathMCQA, CT-US1, MRI-US1, internal histopathology, internal dermatology and EyePACS. Other training and evaluation datasets are publicly available.

## Code availability

The models have been released openly, with information and details at the Google Health AI Developer Foundations site at https://goo.gle/hai-def and https://goo.gle/medgemma. These public sites also include code examples for running, fine-tuning and evaluating MedGemma.
