# Supplementary material for: An open vision-language model for diverse medical applications
Source: Nat Med. 2026 Oct 6;32(10):3695–704. doi: 10.1038/s41591-026-04626-w (PMC13668717; doi:10.1038/s41591-026-04626-w)
Supplement: Supplementary file 1 [file 41591_2026_4626_MOESM1_ESM.pdf]

nature medicine

Supplementary information https://doi.org/10.1038/s41591-026-04626-w

# An open vision-language model for diverse medical applications

In the format provided by the authors and unedited

## A. MedGemma Evaluations

MedGemma was evaluated and compared with other models on five types of medical tasks: text question-answering, image classification, visual question answering, chest X-ray (CXR) report generation, and agentic behavior. We additionally validated MedGemma on several general purpose (non-medical) benchmarks. A high-level overview of tasks and datasets is provided in Table 1 of the main text and additional details on each task are below.

### A.1. General evaluation approach

**Evaluation parameters:** Unless reported otherwise, all evaluations that we performed consisted of a single inference run per example. For MedGemma evaluations, a temperature of 0.0 was used on medical benchmarks and the default temperature was used on non-medical benchmarks. For evaluations of all other models, on all datasets, each model's default temperature and top-k were used. Due to data privacy and license terms, only publicly available datasets were used in evaluating models involving public APIs (e.g. OpenAI models). In cases where existing literature with performance metrics was available, those values were used and noted (with inclusion criteria for external models described below). For generalist models, we found that giving them a persona in the system message, such as "You are a helpful medical assistant" or "You are a helpful radiology assistant" could improve their performance; we thus added these messages into the evaluation prompts. Detailed prompt usages can be found in Supplementary Table 15 and Supplementary Table 16. As the DeepSeek-R1 model [1] is a text-only model, it was only evaluated on text benchmarks.

**Inclusion of previously published model performance data:** Where comparisons to other models were made, we restricted inclusion to models that met the following criteria: a publicly accessible model card (e.g., via Hugging Face or an institutional website); clear and verifiable attribution, including the name and contact information of the responsible individual or institution; explicit licensing terms governing its use; at least one associated technical report or publication for the current or a prior version of the model. These inclusion criteria were chosen to help ensure accountability, transparency, and adherence to sound machine learning practices, such as avoiding test dataset leakage. Additionally, for visual question answering (VQA) comparisons, we only include zero-shot generative evaluations for the most direct and meaningful comparisons (thus excluding few-shot or discriminative, embedding-based approaches for VQA).

### A.2. Medical text question-answering

For evaluation of closed ended medical and health related capabilities, we used the official, publicly available test splits for MedQA, MedMCQA, PubMedQA, MMLU medical subcategories, AfriMed-QA, and MedXpertQA. AfriMed-QA includes a mix of closed and open questions though we limited evaluations to only the closed multiple choice questions (MCQs). No data from MedXpertQA was used in model training, so it is considered an out-of-distribution (OOD) benchmark. Beyond the results on text question answering shown in Table 2 of the main text, we show further results with confidence intervals for model and dataset combinations on which we internally calculated performance in Supplementary Table 1.

To evaluate quality of responses to open ended medical question answering we used a collection of 130 medical content questions as described previously [3]. Responses to each question were reviewed by three U.S. board certified physicians, with a focused task of rating overall factual accuracy of the response. The possible ratings included accurate, inaccurate, unsupported, or unable to rate. The majority vote rating was used for the analysis provided. If no majority, the "worst case" rating was used.

**Supplementary Table 1 | Accuracy on text-only medical benchmarks with confidence intervals.** Accuracy numbers with confidence intervals are shown only for models and datasets that we calculated (not including externally reported results). Metrics for MedQA correspond to the original 4 option test set. 95% confidence intervals were generated via bootstrap resampling with 5000 samples except where otherwise specified.

| Model | **Open** † | MedQA | MedMCQA | PubMedQA | MMLU Anatomy | MMLU Clinical Knowl. | MMLU College Biology | MMLU College Medicine | MMLU Medical Genetics | MMLU Prof. Medicine | MMLU Virology | AfriMed |
|---|---|---|---|---|---|---|---|---|---|---|---|---|
| **Small Models** | | | | | | | | | | | | |
| MedGemma 4B | ✓ | 64.1 [61.4, 66.5] | 55.7 [54.9, 56.5] | 73.4 [69.6, 77.4] | 59.3 [51.1, 67.6] | 71.3 [65.7, 76.9] | 70.8 [63.2, 78.1] | 65.3 [57.7, 72.3] | 83.0 [75.3, 90.0] | 76.8 [71.7, 81.8] | 53.0 [45.5, 60.5] | 52.0 [32.0, 72.0] |
| Gemma 3 4B | ✓ | 52.4 [49.7, 55.1] | 45.7 [44.2, 47.2] | 68.4 [64.4, 72.4] | 54.1 [45.7, 62.4] | 70.2 [64.5, 75.5] | 77.8 [70.9, 84.5] | 63.6 [56.2, 70.5] | 74.0 [65.4, 82.5] | 65.4 [59.8, 71.1] | 42.8 [35.2, 50.4] | 48.0 [28.0, 68.0] |
| MedGemma 27B Text (with test-time scaling [2]) | ✓ | 86.2 [84.4, 88.1] | 72.4 [71.0, 73.8] | 76.2 [72.4, 79.8] | 83.7 [77.5, 89.8] | 86.0 [81.6, 90.0] | 96.5 [93.2, 99.3] | 86.1 [81.0, 91.1] | 97.0 [93.0, 100.0] | 93.4 [90.3, 96.2] | 53.6 [46.0, 61.3] | 84.0 [68.0, 96.0] |
| Gemma 3 27B | ✓ | 74.9 [72.5, 77.4] | 62.6 [61.1, 64.0] | 73.4 [69.6, 77.2] | 74.8 [67.3, 81.8] | 86.0 [81.8, 90.0] | 93.8 [89.4, 97.3] | 78.6 [72.2, 84.7] | 91.0 [85.1, 96.2] | 85.7 [81.5, 89.7] | 51.2 [43.6, 59.0] | 68.0 [48.0, 84.0] |
| **Large Models** | | | | | | | | | | | | |
| DeepSeek-R1 | ✓ | 91.0 [89.4, 92.6] | 78.8 [77.6, 80.1] | 77.2 [73.6, 80.8] | 91.1 [85.9, 95.6] | 91.7 [88.2, 94.9] | 98.6 [96.5, 100.0] | 90.8 [86.2, 94.9] | 99.0 [96.6, 100.0] | 95.6 [93.0, 97.8] | 56.0 [48.4, 63.7] | 92.0 [74.0, 99.0]* |
| Gemini 2.5 Flash | × | 91.5 [89.9, 93.0] | 79.7 [78.0, 80.5] | 76.2 [73.0, 80.2] | 91.1 [86.0, 95.6] | 91.3 [87.8, 94.5] | 96.5 [93.2, 99.3] | 85.0 [79.4, 90.1] | 100.0 [100.0, 100.0] | 97.1 [94.8, 98.9] | 56.0 [48.6, 63.3] | 84.0 [68.0, 96.0] |
| Gemini 2.5 Pro | × | 93.9 [92.5, 95.1] | 83.6 [82.5, 84.7] | 75.8 [71.2, 78.6] | 91.1 [85.9, 95.5] | 91.7 [88.1, 95.0] | 98.6 [96.4, 100.0] | 89.0 [84.0, 93.5] | 96.0 [91.8, 99.1] | 96.3 [94.0, 98.5] | 56.0 [48.5, 63.2] | 84.0 [68.0, 96.0] |
| GPT 4o | × | 91.0 [89.5, 92.6] | 76.0 [74.8, 77.3] | 78.4 [74.8, 81.8] | 87.4 [81.2, 92.7] | 89.4 [85.5, 93.0] | 94.4 [90.5, 97.9] | 87.9 [82.9, 92.4] | 98.0 [94.8, 100.0] | 94.1 [91.2, 96.8] | 58.4 [50.6, 65.8] | 80.0 [59.3, 93.2]* |
| GPT o3 | × | 93.3 [91.9, 94.7] | 83.9 [82.7, 85.0] | 80.8 [77.2, 84.2] | 92.6 [87.8, 96.7] | 94.7 [91.8, 97.3] | 99.3 [97.7, 100.0] | 90.2 [85.5, 94.4] | 100.0 [100.0, 100.0] | 96.7 [94.5, 98.6] | 56.0 [48.3, 63.9] | 84.0 [68.0, 96.0] |

* 95% confidence intervals computed via Clopper-Pearson binomial confidence interval

† Open-weight models

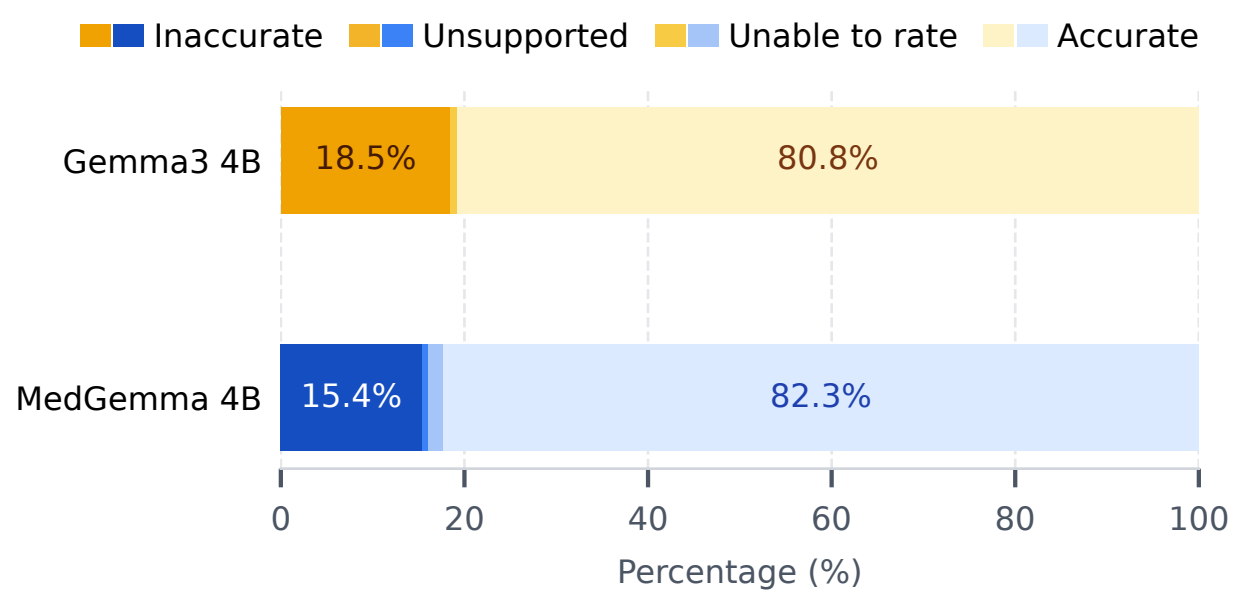


**Supplementary Figure 1 | Factuality evaluation of responses to open-ended medical content questions.** Gemma 3 4B and MedGemma 4B responses to open-ended questions (n=130 from MultiMedQA) were evaluated by three physicians per question to provide ratings on overall factual accuracy of responses. Values represent majority vote ratings. MedGemma 4B demonstrated a 17% relative reduction in factually inaccurate responses (15.4% vs. 18.5% for Gemma 3 4B).

### A.3. Medical image classification

We evaluated medical image classification on three public chest X-ray datasets, as well as private datasets of dermatology, histopathology and retina images. We measured classification performance using accuracy or macro F1, depending on the dataset, targeting common findings or diagnoses in each modality.

**Chest X-rays:** Prediction accuracy was evaluated for five conditions in the MIMIC-CXR [4–6] and CheXpert [7] datasets: atelectasis, cardiomegaly, consolidation, edema, and pleural effusion. Accuracy was evaluated for three conditions in the ChestX-ray14 (CXR14) [8] dataset: lung opacity, pneumothorax, and fracture. We used the original condition labels from CheXpert. For MIMIC-CXR, we report performance on two different versions of the test data: (1) using radiologist-adjudicated labels with missing and uncertain labels excluded, as in Yang *et al.* [9] and (2) using original labels from Johnson *et al.* [10] with missing and uncertain labels considered to be negative, with the same set of test cases as reported in Hyland *et al.* [11]. For the CXR14 data set, radiologist-adjudicated labels as described in Majkowska *et al.* [12] were used for evaluation. Evaluations on MIMIC-CXR were not performed with the OpenAI o3 model due to data privacy considerations.

**Dermatology:** "US-Derm MCQA" [13] is an internal, de-identified dataset consisting of one image per patient from 1996 patients who were referred to tele-dermatologists by primary care physicians in the United States. There are 136 different skin conditions represented across the images, with ground truth diagnoses provided by dermatologists based on the images and metadata. We converted this dataset into a multiple choice question format where the associated reference condition is included among three other randomly assigned condition labels from the same dataset (four options total per MCQ).

**Histopathology:** "Path MCQA" is an internal dataset of 450 patches extracted from 354 unique whole slide images associated with specimens from breast cancer, lung cancer, prostate cancer, lymph nodes, and cervical biopsies. These represent test splits (by patient) from several different data sources. Patches comprise magnifications of 5x, 10x, and 20x (2, 1, and 0.5 microns per pixel). A single, multiclass labeling task for each patch was formulated as a multiple choice question, with four to nine possible options per question depending on the tissue type and labeling task associated with the image. These questions focus on identification and grading for breast cancer, prostate cancer, and cervical dysplasia as well as lung cancer histologic classification and histologic sub-typing. Ground truth labels were obtained via annotations provided as region-level labels by US board-certified pathologists [14–17].

**Ophthalmology:** We used the de-identified 45-degree retina imagery dataset from EyePACS [18] as described previously [9]. We evaluated one image per patient from 3161 patients on clinically-determined 5-class diabetic retinopathy (DR) severity labels, with each task formulated as a multiple choice question with five options: none, mild, moderate, severe or proliferative DR.

### A.4. Medical visual question-answering

We measured visual question-answering performance on the radiology SLAKE and VQA-RAD data sets using the average tokenized F1 metric across open and closed QAs, as well as accuracy on the subset of yes/no questions. For SLAKE, we used the default train/test split, and for VQA-RAD, we used splits from Yang *et al.* [9][1] to avoid the train/test image contamination present in the original splits.

[1] https://github.com/Google-Health/google-health/blob/master/data_splits/

### A.5. Chest X-ray report generation

The pretrained checkpoint of MedGemma 4B was used to generate radiology reports from the MIMIC-CXR test set. The pretrained model was chosen here rather than the post-trained model due to the sensitivity to reporting style of metrics like RadGraph F1 [19]. The pretrained model could better follow the style of MIMIC-CXR, as MIMIC-CXR reports are used in training, while the post-trained model conformed more closely to the original Gemma 3 style in terms of report generation.

We measured accuracy of chest X-ray report generation on the MIMIC-CXR dataset by comparing MedGemma-generated reports to the original radiologist reports for both impression and findings using the RadGraph F1 metric [19] on the 912 image set used in Tanno *et al.* [20] and Yang *et al.* [9].

We also performed a human expert evaluation on the same 306-case image set as in Yang *et al.* [9] with a US board-certified cardiothoracic radiologist to evaluate both the original and MedGemma-generated reports with respect to the associated chest X-ray image. The evaluation task compared the reports on a five-point scale, as originally described in Yang *et al.* [9] and shown in Supplementary Table 10. Although the reviewer was asked to remain neutral in their evaluation, they were not blinded to which report was from the original radiologist vs. from the AI system. This evaluation complements the automated RadGraph-based evaluation as it can both distinguish between major and minor issues as well as account for scenarios in which the original MIMIC-CXR report contains errors or omissions.

### A.6. Medical agentic behavior

To provide insights into the capabilities of MedGemma in more complex environments, we measured the ability of MedGemma to operate in an agentic setting. We evaluated on AgentClinic [21], which positions MedGemma in the role of a “physician agent” in a simulated clinical environment. Solving tasks in AgentClinic required the model to perform dialogue-driven patient history taking, ordering and interpreting medical exams, and operating under incomplete information in order to accurately provide a final diagnosis. We evaluated on 415 simulated cases using the text-only environments, AgentClinic-MedQA (215 cases) and AgentClinic-MIMIC-IV (200 cases), which are derived from MedQA [22] and MIMIC-IV [23] respectively.

### A.7. General purpose benchmarks

Given the limitations that many specialized medical models exhibit when faced with non-medical tasks, we also evaluated possible tradeoffs of specialization by evaluating on the MMLU Pro, Global MMLU Lite, and MMMU benchmarks. Performance on the MMMU benchmark is reported on the validation set as the public test set does not include answers.

## B. MedSigLIP Evaluations

MedSigLIP was evaluated both on zero-shot classification performance and linear probe classification performance (also referred to as data-efficient classification). Zero-shot classification performance was assessed as a measure of baseline performance, and linear probe performance was assessed to gauge performance on target conditions after additional training with logistic regression.

Evaluation datasets spanned four modalities: chest X-ray, dermatology, ophthalmology and histopathology. For chest X-ray, we followed the evaluation datasets and framework in ELIXR [24]. For linear probing, we utilized the same data from CheXpert and CXR14 and evaluated on seven findings (atelectasis, cardiomegaly, airspace opacity, fracture, pneumothorax, consolidation, pleural effusion, and pulmonary edema). For zero-shot, we compared with ELIXR on the 13 positive findings from the CheXpert test set. For dermatology, we evaluated US-Derm MCQA. The US-Derm MCQA test set has 79 dermatological conditions that overlap with the classification labels from the training dataset, thus we pick this 79-condition subset (1612 patients) for our vision based classification and zero-shot evaluation. For ophthalmology, we used the EyePACS test dataset. For histopathology, patches were extracted from whole slide images across a variety of tissue types and tasks as in Yang *et al.* [9] and these patches were treated as individual images. See Supplementary Table 11 for a description of the detailed classes for the evaluations. Additionally, the eva framework [25] was used with default parameters and dataset splits (patch resizing to 448x448 pixels and image normalizations to 0.5 for SigLIP encoder) to further evaluate MedSigLIP on the BACH [26], BreakHis [27], PatchCamelyon [28], and CRC-100k datasets [29].

**Zero-shot evaluation approach and metrics:** For each class within each condition, one or more text prompts were used to represent the class. When there were multiple prompts for the same class, the text embeddings for each prompt were averaged together to obtain a single embedding per class. Cosine similarity was then calculated between the image embedding and text embeddings for the class options, softmax was applied to output scores, and the AUC (area under receiver operating characteristic curve) was calculated. In the case of multiclass conditions, 1-vs-all AUC was calculated. For dermatology, zero-shot prompts are directly their condition names. Prompts for ophthalmology, chest X-ray, histopathology are provided in Supplementary Table 12, Supplementary Table 13, and Supplementary Table 14.

**Linear probe evaluation approach and metrics:** Linear probe (data efficient) classification was assessed by extracting image embeddings from MedSigLIP (without use of the text encoder) and training a logistic regression using the SAGA solver [30] on the train set embeddings, with the hyper-parameters chosen on the validation set, then evaluating on the test set.

## C. MedSigLIP Results

Zero-shot and linear probe classification results and evaluation tasks for MedSigLIP and corresponding Health AI Developer Foundations (HAI-DEF) [31] models are summarized below. For the HAI-DEF Derm Foundation and Path Foundation models, zero-shot classification was not possible with the comparator models because of their image-only nature.

On CXR, MedSigLIP is compared to the HAI-DEF CXR Foundation model, which is based on ELIXR [24], with zero-shot results shown in Supplementary Table 4 and linear probe classification results shown in Supplementary Fig. 2. On average, MedSigLIP's zero-shot CXR performance was 2.0% higher than CXR Foundation despite MedSigLIP's lower image resolution (448×448 vs 1280×1280) and multi-domain expertise, suggesting that MedSigLIP can serve as a strong foundation model. Notably, classification of fractures, which has historically proven difficult, improved over ELIXR by 7.1%. Across the 7 findings used for linear probing, MedSigLIP also demonstrated strong performance

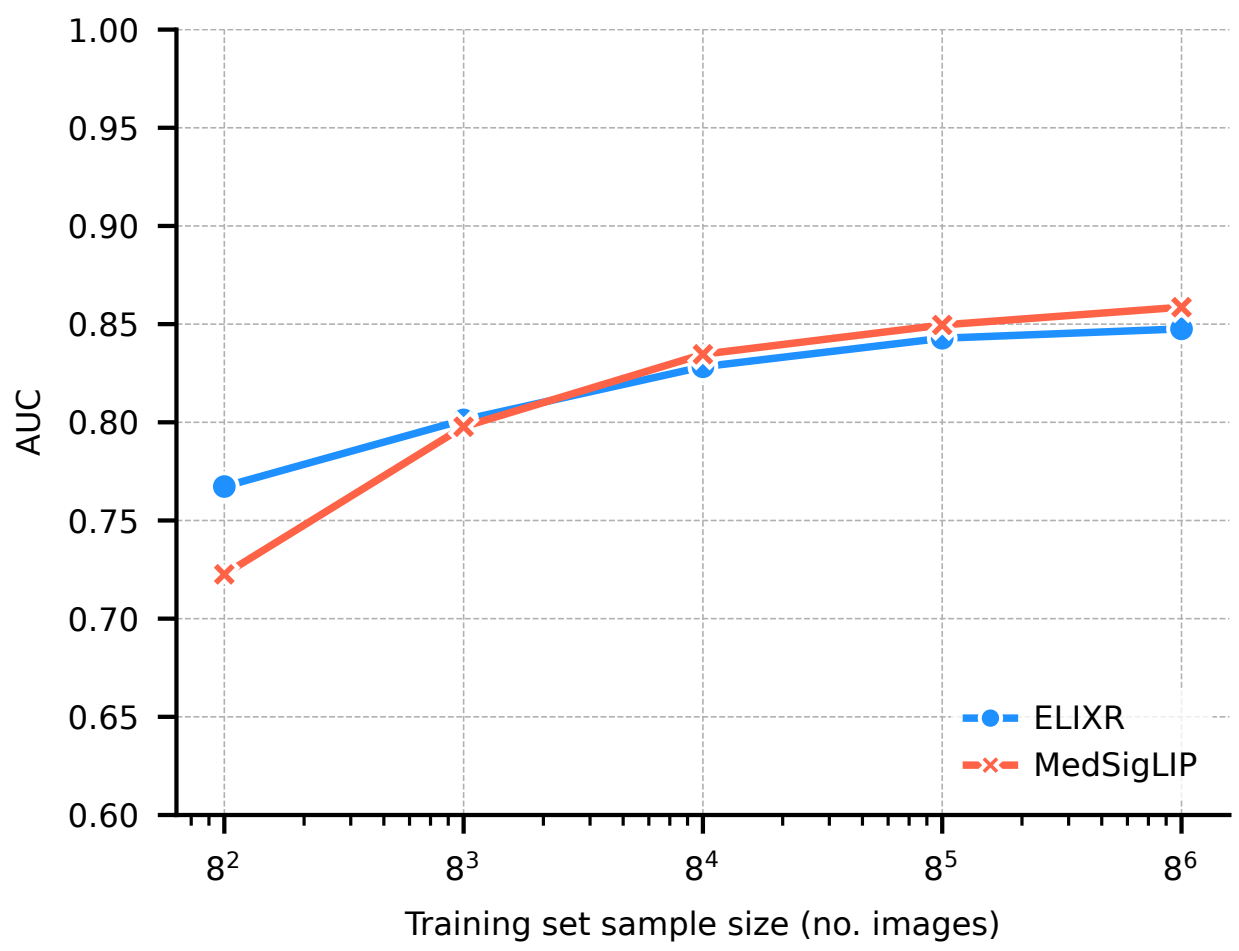


**Supplementary Figure 2 | MedSigLIP data efficient learning results for chest X-ray classification.** Average results for data efficient learning on 7 chest X-ray findings on CheXpert and CXR14 datasets compared to HAI-DEF's CXR Foundation model based on ELIXR [24]. Individual results per condition per dataset can be found in Supplementary Fig. 3 and Supplementary Fig. 4.

when training set sample size is larger or equal to 512 examples (Supplementary Fig. 2).

**Supplementary Table 2 | AUCs for dermatology, ophthalmology, and histopathology findings with MedSigLIP and HAI-DEF image models [31].** HAI-DEF image models include Derm Foundation and Path Foundation for dermatology and histopathology, respectively. Bold values indicate the best results in each section.

| Domain | Finding | N | No. Classes | MedSigLIP Resolution | MedSigLIP Zero-Shot | MedSigLIP Linear Probe | HAI-DEF (image)§ Resolution | HAI-DEF (image)§ Linear Probe |
|---|---|---|---|---|---|---|---|---|
| Dermatology | Skin Conditions | 1612 | 79 | 448x448 | 0.851 | **0.881** | 448×448 | 0.843 |
| Ophthalmology | Diabetic Retinopathy | 3161 | 5 | 448x448 | 0.759 | **0.857** | N/A | N/A |
| Histopathology | Invasive Breast Cancer | 5000 | 3 | 448×448 | 0.933 | 0.930 | 224×224 | **0.943** |
| | Breast NP | 5000 | 3 | | 0.721 | 0.727 | | **0.758** |
| | Breast TF | 5000 | 3 | | 0.780 | 0.790 | | **0.832** |
| | Cervical Dysplasia | 5000 | 3 | | 0.889 | 0.864 | | **0.898** |
| | Prostate Cancer Needle Core Biopsy | 5000 | 4 | | 0.892 | 0.886 | | **0.915** |
| | Radical Prostatectomy | 5000 | 4 | | 0.896 | 0.887 | | **0.921** |
| | TCGA Study Types | 5000 | 10 | | 0.922 | **0.970** | | 0.964 |
| | Tissue Types | 5000 | 16 | | 0.930 | **0.972** | | 0.947 |
| **Average** | | | | | 0.870 | 0.878 | | **0.897** |

§ Prior reported results from HAI-DEF image-only foundation models [31].

Dermatology, ophthalmology and histopathology results are summarized in Supplementary Table 2. For dermatology, both MedSigLIP zero-shot and linear probes outperformed linear probes with Derm Foundation on the task of distinguishing between 79 skin conditions. For ophthalmology, on the 5-class task of classifying diabetic retinopathy (none, mild, moderate, severe, and proliferative), linear probe performance exceeded zero-shot performance by 9.8%, though no HAI-DEF model currently exists for comparison. For histopathology, linear probing achieved a modest improvement over zero-shot classification using MedSigLIP, and performing close to Path Foundation which has linear probe performance that is 1.9% better on average. Additional analysis of MedSigLIP using the eva framework for histopathology embedding evaluation [25] is shown in Supplementary Table 3. MedSigLIP showed reasonable performance despite patch resizing, albeit with some room for improvement to match state of the art vision encoders specialized for histopathology.

MedSigLIP offers strong baseline performance across a variety of medical domains as a single model and generally does well with zero-shot classification even when compared to linear probes with dedicated, domain-specific models. Further training with task-specific images and logistic regression

**Supplementary Table 3 | MedSigLIP performance compared to other pathology embeddings models using a shared framework.** Balanced accuracy for linear probing using MedSigLIP was calculated using the eva framework [25] with default settings and data splits. Bold values indicate the best results in each section.

| Model | BACH | BreakHis | PatchCamelyon test | CRC-100k |
|---|---|---|---|---|
| MedSigLIP | 0.764 | 0.789 | 0.878 | 0.940 |
| H-opitmus 0§ | 0.759 | 0.801 | **0.943** | 0.955 |
| UNI§ | 0.785 | 0.785 | 0.937 | 0.944 |
| Virchow2§ | **0.883** | **0.821** | 0.938 | **0.967** |

§ Prior reported results

**Supplementary Table 4 | Zero-shot AUCs for chest X-ray findings comparing MedSigLIP with HAI-DEF CXR Foundation [24, 31].** Bold values indicate the best results in each section.

| Domain | Finding | N | No. Classes | MedSigLIP | | HAI-DEF (CXR Foundation)§ | |
|---|---|---|---|---|---|---|---|
| | | | | Resolution | Zero-Shot | Resolution | Zero-Shot |
| CXR | Enlarged Cardiomediastinum | 518 | 2 | 448×448 | **0.858** | 1280×1280 | 0.800 |
| | Cardiomegaly | | | | **0.904** | | 0.891 |
| | Lung Opacity | | | | **0.931** | | 0.888 |
| | Lung Lesion | | | | **0.822** | | 0.747 |
| | Consolidation | | | | **0.880** | | 0.875 |
| | Edema | | | | **0.891** | | 0.880 |
| | Pneumonia | | | | 0.864 | | **0.881** |
| | Atelectasis | | | | **0.836** | | 0.754 |
| | Pneumothorax | | | | **0.862** | | 0.800 |
| | Pleural Effusion | | | | 0.914 | | **0.930** |
| | Pleural Other | | | | 0.650 | | **0.729** |
| | Fracture | | | | **0.708** | | 0.637 |
| | Support Devices | | | | 0.852 | | **0.894** |
| **Average** | | | | | **0.844** | | 0.824 |

§ Prior reported results

can yield stronger results.

## D. Additional Training Data Details

**Supplementary Table 5 | Distribution of data for vision encoder training.**

| Category | % of total medical imaging data |
|---|---|
| Radiology | 60.95% |
| Pathology | 2.15% |
| Ophthalmology | 28.7% |
| General Medical (PMC-OA) | 6.05% |
| Dermatology | 2.15% |

**Supplementary Table 6 | Distribution of data for multimodal decoder training.**

| Category | % of Total |
|---|---|
| Radiology | 4.50% |
| Pathology | 3.02% |
| Ophthalmology | 2.59% |
| General Medical (PMC-OA) | 1.85% |
| Dermatology | 0.13% |
| General Non-Medical (Gemma PT mixture) | 87.92% |

**Supplementary Table 7** | **Overview of histopathology datasets.**

| Dataset Name | Description |
| --- | --- |
| Pathology dataset 1 | De-identified dataset of histopathology H&E whole slide images created in collaboration with an academic research hospital and biobank in Europe. Comprises de-identified colon, prostate, and lymph nodes. |
| Pathology dataset 2 | De-identified dataset of lung histopathology H&E and IHC whole slide images created by a commercial biobank in the United States. |
| Pathology dataset 3 | De-identified dataset of prostate and lymph node H&E and IHC histopathology whole slide images created by a contract research organization in the United States. |
| Pathology dataset 4 | De-identified dataset of histopathology whole slide images created in collaboration with a large, tertiary teaching hospital in the United States. Comprises a diverse set of tissue and stain types, predominantly H&E. |
| TCGA | The Cancer Genome Atlas: De-identified dataset of H&E Histopathology WSIs across multiple cancer types and multiple hospital sites in the United States. These data are made available by the TCGA Research Network: https://www.cancer.gov/tcga and accessible via Genomic Data Commons (GDC). |
| CAMELYON16 | De-identified dataset of H&E Histopathology WSIs representing lymph nodes and metastatic breast cancer to lymph nodes [32]. |

**Supplementary Table 8 | Examples of histopathology captions and patches for training.** Data from 17 unique tissue types and 12 unique cancer types from prior work [33] were used in training. Multi-class annotation masks were used for both sampling image patches from whole-slide images as well as generating captions. Patches of size 896 × 896 were sampled from whole slide images in a class-balanced manner. For each of 11 multi-class annotation "tasks", up to 1,000,000 image patches were sampled for three different magnification levels (2, 1, and 0.5 microns-per-pixel). Multiple captions per class for each task were generated (Gemini 1.5 Pro) and then manually reviewed to ensure an appropriate level of detail and accuracy, resulting in 5–7 captions per class across tasks.

| Task or Topic | Example Captions |
|---|---|
| Breast cancer detection in lymph nodes | Microscopic view of a lymph node with infiltrating malignant epithelial cells consistent with metastatic breast carcinoma.<br>Lymph node section on histopathology demonstrating features of metastatic carcinoma, likely originating from the breast.<br>Region of a lymph node on histopathology, showing predominantly lymphocytes and immune cells. |
| Histologic subtyping for lung adenocarcinoma | H&E histopathology image demonstrating acinar subtype lung adenocarcinoma with glandular formation.<br>Cribriform pattern in lung adenocarcinoma with closely packed, back-to-back glands lacking an organized pattern.<br>Microscopic view of lung adenocarcinoma showing tumor cells spreading along the preexisting alveolar architecture, consistent with the lepidic subtype. |
| Prostate Gleason grading | Microscopic view of a prostate biopsy with discrete, uniformly sized and shaped glands consistent with Gleason pattern 3 prostate cancer.<br>Prostate biopsy image highlighting areas of fused glands or poorly formed glands, consistent with Gleason pattern 4 carcinoma.<br>Microscopic view of a prostate tissue with discrete, uniformly sized and shaped glands consistent with Gleason pattern 3 prostate cancer. |
| Breast cancer nuclear pleomorphism grading | Microscopic view of invasive breast carcinoma showing moderate nuclear atypia, including enlarged nuclei and prominent nucleoli (nucleopleomorphism score 2).<br>Focus on infiltrating tumor cells within an H&E stained image, demonstrating bland nuclear features suggestive of low-grade invasive breast carcinoma.<br>H&E histopathology image demonstrating invasive breast carcinoma with high-grade nuclear features (nucleopleomorphism score 3). |
| Breast cancer tubule formation grading | This microscopic view demonstrates a predominance of well-defined glandular structures, indicating a tubule formation score of 1 in this breast carcinoma.<br>The presence of some discernible tubules, alongside regions of less-defined glandular architecture, indicates a tubule formation score of 2.<br>A tubule formation score of 3 is evident within this H&E stained image, where the invasive carcinoma shows a scarcity of well-defined glandular structures. |
| Cervical dysplasia grading | H&E stained image of a cervical biopsy demonstrating features of cervical intraepithelial neoplasia grade 1 (CIN 1), including nuclear atypia in the lower third of the epithelium.<br>Microscopic view of a cervical biopsy with CIN 1, showing enlarged nuclei and increased nuclear-to-cytoplasmic ratio in the basal layer of the epithelium.<br>H&E histopathology of a cervical biopsy focusing on high-grade dysplasia, showing prominent nuclear abnormalities and disruption of the normal epithelial architecture. |

## E. MedGemma variants and summarized results

### E.1. Note on model variants:

As noted in the methods, the multimodal models, MedGemma 4B and MedGemma 27B, utilized the same overall training recipe, while MedGemma 27B Text leveraged the post-training stage alone. Also, relative to MedGemma 4B, training of MedGemma 27B included two additional training datasets: EHRQA (details in Supplementary Section I) and Chest ImaGenome [6, 34]. As such, MedGemma 27B essentially provides all of the same capabilities as MedGemma 4B with improved language capabilities due to its larger size as well as improved EHR understanding and improved anatomy localization for chest X-rays.

### E.2. Summarized results across model variants:

For ease of reference of performance on different benchmarks and tasks across variants, the results are summarized in Supplementary Table 9.

**Supplementary Table 9 | Summarized results for MedGemma models across tasks.**

| Task | Metric | MedGemma 4B | MedGemma 27B Text | MedGemma 27B |
|---|---|---|---|---|
| Text evaluation | | | | |
| MedQA (4-op) | Accuracy | 64.1 | 86.2 | 85.3 |
| MedMCQA | Accuracy | 55.7 | 72.4 | 70.2 |
| PubMedQA | Accuracy | 73.4 | 76.2 | 77.2 |
| MMLU Med | Accuracy | 70.0 | 87.0 | 86.2 |
| MedXpertQA (text only) | Accuracy | 14.2 | 25.7 | 23.7 |
| AfriMed-QA | Accuracy | 52.0 | 84.0 | 72.0 |
| Electronic health record information retrieval | | | | |
| EHRQA | Accuracy | 67.6 | 86.3 | 90.5 |
| Medical image classification | | | | |
| MIMIC CXR | Average F1 for 5 conditions | 88.9 | N/A | 90.0 |
| CheXpert CXR | Average F1 for 5 conditions | 48.1 | N/A | 49.9 |
| CXR14 | Average F1 for 3 conditions | 50.1 | N/A | 45.3 |
| DermMCQA | Accuracy | 71.8 | N/A | 71.7 |
| PathMCQA | Accuracy | 69.8 | N/A | 71.6 |
| Eyepacs | Accuracy | 64.9 | N/A | 75.3 |
| Visual question answering | | | | |
| SlakeVQA | Tokenized F1 | 72.3 | N/A | 70.3 |
| VQA-RAD | Tokenized F1 | 49.9 | N/A | 46.7 |
| Knowledge and reasoning | | | | |
| MedXpertQA (text + MM) | Accuracy | 18.8 | N/A | 26.8 |
| Report generation | | | | |
| MIMIC CXR | Radgraph F1 | 29.5* | N/A | 27.0* |
| Localization of anatomical regions | | | | |
| Chest ImaGenome | Mean IoU | 3.1 | N/A | 16.0 |

* Results from the pretrained checkpoints.

## F. Manual Evaluation of Radiology reports

Supplementary Table 10 provides the detailed rubric definitions provided to the radiologist for scoring the generated CXR reports.

**Supplementary Table 10 | Human evaluation rubric comparing AI generated radiology reports to original reports.**

| Label | Definition |
|---|---|
| AI » Original Report | AI report captures key clinically relevant findings that are not found in original report. AI report would result in correct patient management and original report would not. |
| AI > Original Report | AI report captures more relevant findings, but both would result in the same correct patient management. |
| AI ~ Original Report | Both reports capture similar findings in the image and would result in correct patient management. |
| AI < Original Report | Original report captures more relevant findings, but both would result in the same correct patient management. |
| AI « Original Report | Original report captures key clinically relevant findings that are not found in AI report. Original report would result in correct patient management and AI report would not. |
| X | Neither report would result in correct patient management. |

## G. Evaluation Prompts

For evaluating MedSigLIP zero-shot tasks, Supplementary Table 11, Supplementary Table 12, Supplementary Table 13 and Supplementary Table 14 list the prompts used.

For evaluating MedGemma, the default temperature of 0.0 was used. All other models made use of their default system temperature. Two different system prompts were used in providing results for other LLMs directly evaluated:

- *You are a helpful radiology assistant.* For radiology-based tasks.
- *You are a helpful medical assistant.* For all other medical tasks.

Per-dataset prompts used are shown in Supplementary Table 15 and Supplementary Table 16.

**Supplementary Table 11 | Classes for the findings on which MedSigLIP was evaluated.**

| Domain | Finding | Num Classes | Class Names |
|---|---|---|---|
| Radiology | CXR | 2 | Positive/negative for Cardiomegaly, Consolidation, Edema, Enlarged cardiomediastinum, Fracture, Lung lesion, Lung opacity, Pleural effusion, Pleural other, Pneumonia, Pneumothorax,Support devices. |
| Histopathology | Invasive Breast Cancer | 3 | Benign, Invasive Carcinoma, DCIS |
| | Breast NP | 3 | NP1, NP2, NP3 |
| | Breast TF | 3 | TF1, TF2, TF3 |
| | Cervical Dysplasia | 3 | Normal, CIN Grade 1, CIN Grade 2+ |
| | Prostate Cancer Needle Core Biopsy | 4 | Benign, GP3, GP4, GP5 |
| | Radical Prostatectomy | 4 | Benign, GP3, GP4, GP5 |
| | TCGA Study Types | 10 | BLCA, BRCA, COAD, HNSC, KIRC, LIHC, LUAD, LUSC, OV, STAD |
| | Tissue Types | 16 | Appendix, Breast, Cervix, Colon, Fallopian Tube, Gallbladder, Liver, Lymph node, Ovary, Placenta, Prostate, Skin, Thyroid, Upper GI, Uterus, Vas deferens |
| Dermatology | Skin Conditions | 79 | Acanthosis nigricans, Acne, Acne keloidalis, Actinic Keratosis, Allergic Contact Dermatitis, Alopecia Areata, Amyloidosis of skin, Androgenetic Alopecia, Angiokeratoma of skin, Atypical Nevus, Basal Cell Carcinoma, Bullous Pemphigoid, Burn of skin, Candida, Clavus, Comedone, Condyloma acuminatum, Cutaneous T Cell Lymphoma, Cutaneous lupus, Cutaneous sarcoidosis, Cyst, Dermatofibroma, Drug Rash, Eczema, Erythema ab igne, Erythema multiforme, Folliculitis, Folliculitis decalvans, Fordyce spots, Granuloma annulare, Hemangioma, Herpes Zoster, Hidradenitis, Idiopathic guttate hypomelanosis, Infected skin lesions, Inflicted skin lesions, Insect Bite, Intertrigo, Irritant Contact Dermatitis, Keratosis pilaris, Knuckle pads, Lentigo, Lichen Simplex Chronicus, Lichen planopilaris, Lichen planus/lichenoid eruption, Lichen sclerosus, Lipodermatosclerosis, Lipoma, Livedo reticularis, Melanocytic Nevus, Melanoma, Melasma, Milia, Nevus sebaceous, O/E - ecchymoses present, Onychomycosis, Perioral Dermatitis, Photodermatitis, Pigmented purpuric eruption, Pityriasis lichenoides, Pityriasis rosea, Post-Inflammatory hyperpigmentation, Prurigo nodularis, Psoriasis, Pyoderma Gangrenosum, Pyogenic granuloma, Rosacea, SCC/SCCIS, SK/ISK, Scabies, Scar Condition, Seborrheic Dermatitis, Skin Tag, Stasis Dermatitis, Tinea, Tinea Versicolor, Urticaria, Verruca vulgaris, Vitiligo |
| Ophthalmology | Diabetic Retinopathy (DR) | 5 | No DR, Mild DR, Moderate DR, Severe DR, Proliferative DR |

**Supplementary Table 12 | Zero shot prompts for MedSigLIP ophthalmology zero-shot evaluation.**

| Ophthalmology | |
|---|---|
| Finding | Severity |
| Diabetic retinopathy | diabetic retinopathy severity: none.<br>diabetic retinopathy severity: mild.<br>diabetic retinopathy severity: moderate.<br>diabetic retinopathy severity: severe.<br>diabetic retinopathy severity: proliferative. |

**Supplementary Table 13 | Zero shot prompts for MedSigLIP CXR zero-shot evaluation.**

| | Chest X-ray | |
|---|---|---|
| Finding | Condition Absent | Condition Present |
| Atelectasis | no atelectasis<br>no acute cardiopulmonary process | adjacent atelectasis<br>bibasilar atelectasis<br>there is atelectasis |
| Cardiomegaly | heart size is normal<br>cardiac size is within normal limits<br>cardiothoracic ratio within normal limits<br>no acute cardiopulmonary process<br>normal study<br>no evidence of cardiomegaly | mild cardiomegaly<br>moderate cardiomegaly<br>severe cardiomegaly<br>enlarged cardiac silhouette |
| Consolidation | no focal consolidation<br>no acute cardiopulmonary process<br>normal study | alveolar consolidation<br>densely consolidated<br>lobe consolidation<br>airspace consolidation<br>bibasilar consolidations<br>suggestive of consolidation |
| Edema | no pulmonary edema<br>no acute cardiopulmonary process<br>normal study | mild pulmonary edema<br>moderate pulmonary edema<br>severe pulmonary edema |
| Enlarged cardiomediastinum | no acute cardiopulmonary process<br>cardiomediastinal silhouette is normal | widened cardiomediastinum |
| Fracture | no acute cardiopulmonary process<br>normal study | rib fractures<br>rib fracture |
| Lung lesion | no acute cardiopulmonary process | lytic lesion<br>cavitary lesion<br>parenchymal lesion |
| Lung opacity | no focal opacity<br>lung volumes are normal<br>normal study<br>normal lung volumes<br>lungs are clear<br>no focal consolidation<br>no evidence of airspace consolidation<br>no infiltrate<br>no airspace opacity<br>pulmonary parenchyma is clear | bilateral opacities<br>basal opacity<br>opacification is present<br>bibasilar opacities<br>increased opacification<br>consolidative opacity<br>parenchymal opacities<br>airspace opacification cannot be excluded<br>there is airspace opacification |
| Pleural effusion | no evidence of pleural effusion<br>no acute cardiopulmonary process<br>normal study | left pleural effusion<br>right pleural effusion<br>bilateral pleural effusions |
| Pleural other | no acute cardiopulmonary process | blunting of costophrenic angle<br>pleural thickening |
| Pneumonia | lungs are clear<br>no acute cardiopulmonary process | suggestive of pneumonia |
| Pneumothorax | no pneumothorax<br>no acute cardiopulmonary process<br>normal study | apical pneumothorax |
| Support devices | no acute cardiopulmonary process | monitoring and support devices<br>NG tube<br>ET tube<br>catheter<br>PIC line |

**Supplementary Table 14 | Zero shot prompts for MedSigLIP histopathology zero-shot evaluation.**

| Condition | Class | Prompt |
|---|---|---|
| Invasive Breast Cancer | | |
| | 1 | • region of an HE histopathology image showing benign breast tissue<br>• HE-stained image demonstrating normal breast tissue architecture<br>• histopathology image with breast lobules and ducts consistent with benign breast tissue<br>• microscopic view of breast tissue with regular uniformly spaced glands indicative of benign breast tissue<br>• HE histopathology image showing normal breast stroma and absence of cellular atypia characteristic of benign breast tissue |
| | 2 | • region of an HE histopathology image showing invasive breast carcinoma<br>• HE-stained region demonstrating features of invasive breast carcinoma<br>• histopathology image with areas of dense cellularity and atypical cells consistent with invasive breast carcinoma<br>• microscopic view of breast tissue with disorganized glandular architecture consistent with invasive carcinoma<br>• HE histopathology image with regions of stromal invasion by malignant cells indicating invasive breast carcinoma |
| | 3 | • region of an HE histopathology image showing ductal carcinoma in situ (DCIS)<br>• HE-stained image demonstrating abnormal cells confined within the breast duct<br>• histopathology image: atypical cells within the duct consistent with DCIS<br>• microscopic view of breast tissue showing abnormal cellular proliferation within a duct consistent with DCIS<br>• HE histopathology section with non-invasive intraductal malignant cells suggestive of DCIS |
| Breast NP | | |

(Continued on next page)

(Continued)

| Condition | Class | Prompt |
|---|---|---|
| | 1 | • HE histopathology image demonstrating invasive breast carcinoma with low-grade nuclear features (nucleopleomorphism score 1)<br>• region of invasive breast carcinoma exhibiting relatively uniform nuclei with inconspicuous nucleoli consistent with a low nucleopleomorphism score<br>• microscopic view of invasive breast carcinoma showing limited variation in nuclear shape and size (nucleopleomorphism score 1)<br>• focus on infiltrating tumor cells within an HE-stained image demonstrating bland nuclear features suggestive of low-grade invasive breast carcinoma<br>• HE histopathology section of invasive breast carcinoma with well-formed glands and minimal nuclear atypia indicating a low nucleopleomorphism score |
| | 2 | • HE histopathology image demonstrating invasive breast carcinoma with moderate nuclear pleomorphism (score 2)<br>• region of invasive breast carcinoma exhibiting some variation in nuclear size and shape with some visible nucleoli (nucleopleomorphism score 2)<br>• microscopic view of invasive breast carcinoma showing moderate nuclear atypia including enlarged nuclei and prominent nucleoli (nucleopleomorphism score 2)<br>• focus on tumor cells with increased nuclear irregularity compared to normal breast tissue consistent with a nucleopleomorphism score of 2<br>• HE histopathology section of invasive breast carcinoma with moderately pleomorphic nuclei and discernible nucleoli (nucleopleomorphism score 2) |

(Continued on next page)

(Continued)

| Condition | Class | Prompt |
|---|---|---|
| | 3 | • HE histopathology image demonstrating invasive breast carcinoma with high-grade nuclear features (nucleopleomorphism score 3)<br>• microscopic view of invasive breast carcinoma showing significant nuclear atypia including marked variation in size, shape, and prominent nucleoli (nucleopleomorphism score 3)<br>• region of invasive breast carcinoma exhibiting large nuclei with considerable variation in size and shape as well as large irregular nucleoli (nucleopleomorphism score 3)<br>• HE-stained image highlighting markedly pleomorphic nuclei of tumor cells consistent with high-grade invasive breast carcinoma<br>• focus on an area of invasive breast carcinoma demonstrating significant nuclear abnormalities and variability consistent with a nucleopleomorphism score of 3 |
| Breast TF | | |
| | 1 | • well-formed tubules are visible within this region of invasive breast carcinoma on an HE histopathology image (tubule formation score 1)<br>• this microscopic view demonstrates a predominance of well-defined glandular structures indicating a tubule formation score of 1 in this breast carcinoma<br>• the infiltrating carcinoma cells display preserved tubular architecture resembling normal breast tissue consistent with a tubule formation score of 1<br>• a tubule formation score of 1 is evident on this HE-stained image showcasing numerous well-formed tubules within the breast carcinoma<br>• this region of invasive breast carcinoma depicted on an HE histopathology image exhibits a high degree of tubular differentiation (tubule formation score 1) |

(Continued on next page)

(Continued)

| Condition | Class | Prompt |
|---|---|---|
| | 2 | • this HE histopathology image of invasive breast carcinoma shows a moderate degree of tubule formation (score 2)<br>• while some tubular structures are present this microscopic view highlights a less organized pattern of growth within the breast carcinoma aligning with a tubule formation score of 2<br>• a mix of both well-formed tubules and areas lacking clear glandular structures suggests a tubule formation score of 2 in this breast carcinoma<br>• the presence of some discernible tubules alongside regions of less-defined glandular architecture indicates a tubule formation score of 2<br>• this invasive breast carcinoma displays an intermediate level of tubular differentiation consistent with a tubule formation score of 2 |
| | 3 | • tubular structures are largely absent within this region of invasive breast carcinoma indicating a tubule formation score of 3 on an HE histopathology image<br>• this breast carcinoma demonstrates disorganized growth with minimal gland formation consistent with a tubule formation score of 3<br>• poorly differentiated tumor cells lacking discernible tubules predominate in this microscopic view suggestive of a tubule formation score of 3<br>• a tubule formation score of 3 is evident within this HE-stained image where the invasive carcinoma shows a scarcity of well-defined glandular structures<br>• limited tubule formation characterizes this region of breast carcinoma resulting in a tubule formation score of 3 |
| Cervical Dysplasia | | |
| | 1 | • HE-stained image of a cervical biopsy demonstrating normal squamous epithelium and underlying stroma<br>• microscopic view of a cervical biopsy showing regular stratification of the squamous epithelium without atypia<br>• region of a cervical biopsy with well-defined mature squamous epithelium and unremarkable stroma<br>• cervical biopsy demonstrating normal endocervical glands and adjacent squamous epithelium<br>• HE histopathology of a cervical biopsy showcasing a section of the transformation zone with no pathologic findings |

(Continued on next page)

(Continued)

| Condition | Class | Prompt |
|---|---|---|
| | 2 | • HE-stained image of a cervical biopsy demonstrating features of cervical intraepithelial neoplasia grade 1 (CIN 1) including nuclear atypia in the lower third of the epithelium<br>• microscopic view of a cervical biopsy with CIN 1 showing enlarged nuclei and increased nuclear-to-cytoplasmic ratio in the basal layer of the epithelium<br>• region of a cervical biopsy exhibiting CIN 1 with mild nuclear atypia and koilocytic change (perinuclear halos)<br>• cervical biopsy demonstrating CIN 1 with subtle nuclear abnormalities confined to the lower epithelial layers<br>• HE histopathology of a cervical biopsy with a focus on CIN 1 characterized by slight disorganization of the squamous epithelium |
| | 3 | • HE-stained image of a cervical biopsy demonstrating high-grade dysplasia with significant nuclear atypia and architectural disarray<br>• microscopic view of a cervical biopsy showcasing high-grade squamous intraepithelial lesion (HSIL) with loss of epithelial maturation and cellular disorganization<br>• region of a cervical biopsy exhibiting high-grade dysplasia characterized by increased mitotic activity, nuclear pleomorphism, and loss of normal epithelial polarity<br>• cervical biopsy demonstrating high-grade dysplasia with marked cellular atypia<br>• HE histopathology of a cervical biopsy focusing on high-grade dysplasia showing prominent nuclear abnormalities and disruption of the normal epithelial architecture |
| Prostate Cancer Needle Core Biopsy | | |

(Continued on next page)

(Continued)

| Condition | Class | Prompt |
|---|---|---|
| | 1 | • HE histopathology image showing benign prostate tissue within a prostate specimen<br>• prostate image demonstrating normal prostatic glands and stroma consistent with benign tissue<br>• microscopic view of prostate with well-defined regularly spaced glands and intervening stroma indicative of benign prostatic tissue<br>• focus on a region of benign prostate tissue within a prostate specimen exhibiting a normal glandular architecture and stromal component<br>• HE section of prostate showcasing benign prostatic glands lined by a bilayer epithelium (basal and luminal cells) |
| | 2 | • HE histopathology image demonstrating Gleason pattern 3 prostate cancer within a prostate specimen<br>• prostate image highlighting individual well-formed glands characteristic of Gleason pattern 3 carcinoma<br>• microscopic view of a prostate with discrete uniformly sized and shaped glands consistent with Gleason pattern 3 prostate cancer<br>• focus on Gleason pattern 3 prostate cancer within prostate specimen showing relatively distinct glandular structures with minimal variability<br>• HE section of prostate demonstrating areas of Gleason pattern 3 adenocarcinoma marked by separate glands composed of tumor cells infiltrating between benign glands |
| | 3 | • HE histopathology image demonstrating Gleason pattern 4 prostate cancer within a prostate specimen<br>• prostate image highlighting areas of fused glands or poorly formed glands consistent with Gleason pattern 4 carcinoma<br>• microscopic view of prostate showcasing irregular glandular structures with varying shapes and sizes indicative of Gleason pattern 4 prostate cancer<br>• focus on Gleason pattern 4 prostate cancer within a prostate specimen demonstrating loss of normal glandular architecture and a more disorganized growth pattern<br>• HE section of prostate demonstrating regions of Gleason pattern 4 adenocarcinoma characterized by fused or poorly formed glands |

(Continued on next page)

(Continued)

| Condition | Class | Prompt |
|---|---|---|
| | 4 | • HE histopathology image demonstrating Gleason pattern 5 prostate cancer within a prostate specimen<br>• prostate image highlighting sheets of tumor cells lacking any glandular formation characteristic of Gleason pattern 5 carcinoma<br>• microscopic view of prostate showing solid areas of tumor cells representing Gleason pattern 5 prostate cancer<br>• focus on Gleason pattern 5 prostate cancer within a prostate specimen demonstrating poorly differentiated cells and absence of gland formation<br>• HE section of prostate with regions of Gleason pattern 5 adenocarcinoma characterized by a complete lack of glandular differentiation and presence of solid tumor cell clusters |
| Radical Prostatectomy | | |
| | 1 | • HE histopathology image showing benign prostate tissue<br>• small region from prostate specimen demonstrating normal prostatic glands and stroma consistent with benign tissue<br>• microscopic view of a prostate specimen with well-defined regularly spaced glands and intervening stroma indicative of benign prostatic tissue<br>• focus on a region of benign prostate tissue exhibiting a normal glandular architecture and stromal component<br>• HE section showcasing benign prostatic glands lined by a bilayer epithelium (basal and luminal cells) |
| | 2 | • HE histopathology image demonstrating Gleason pattern 3 prostate cancer<br>• small region of prostate on histopathology highlighting individual well-formed glands characteristic of Gleason pattern 3 carcinoma<br>• microscopic view of a prostate tissue with discrete uniformly sized and shaped glands consistent with Gleason pattern 3 prostate cancer<br>• focus on Gleason pattern 3 prostate cancer showing relatively distinct glandular structures with minimal variability<br>• HE section of prostate tissue demonstrating areas of Gleason pattern 3 adenocarcinoma marked by separate infiltrating glands |

(Continued on next page)

(Continued)

| Condition | Class | Prompt |
|---|---|---|
| | 3 | • HE histopathology image demonstrating Gleason pattern 4 prostate cancer<br>• small region of a prostate on histopathology highlighting areas of fused glands, poorly formed glands, or cribriform architecture consistent with Gleason pattern 4 carcinoma<br>• microscopic view of prostate showcasing irregular glandular structures with varying shapes and sizes indicative of Gleason pattern 4 prostate cancer<br>• focus on Gleason pattern 4 prostate cancer demonstrating loss of normal glandular architecture and a more disorganized growth pattern<br>• HE section of prostate tissue demonstrating regions of Gleason pattern 4 adenocarcinoma characterized by fused or poorly defined glands |
| | 4 | • HE histopathology image demonstrating Gleason pattern 5 prostate cancer<br>• small region of prostate on histopathology highlighting presence of sheets of tumor cells lacking any glandular formation characteristic of Gleason pattern 5 carcinoma<br>• microscopic view of prostate showing solid areas of tumor cells with minimal cellular pleomorphism representing Gleason pattern 5 prostate cancer<br>• focus on Gleason pattern 5 prostate cancer demonstrating poorly differentiated cells and absence of gland formation<br>• HE section of prostate with regions of Gleason pattern 5 adenocarcinoma characterized by a complete lack of glandular differentiation and presence of solid tumor cell clusters |
| The Cancer Genome Atlas (TCGA) Study Types | | |
| | 1 | • microscopic view of bladder specimen demonstrating bladder cancer<br>• microscopic view of bladder specimen demonstrating urothelial cell carcinoma<br>• microscopic view of bladder specimen demonstrating urothelial cancer<br>• microscopic view of bladder specimen demonstrating urinary bladder tumor<br>• microscopic view of bladder specimen demonstrating transitional cell carcinoma |

(Continued on next page)

(Continued)

| Condition | Class | Prompt |
|---|---|---|
| | 2 | • microscopic view of breast specimen demonstrating breast cancer<br>• microscopic view of breast specimen demonstrating invasive breast carcinoma<br>• microscopic view of breast specimen demonstrating carcinoma of the breast |
| | 3 | • microscopic view of colon specimen demonstrating colon cancer<br>• microscopic view of colon specimen demonstrating colorectal cancer<br>• microscopic view of colon specimen demonstrating colon adenocarcinoma |
| | 4 | • microscopic view of head and neck squamous cell carcinoma |
| | 5 | • microscopic view of kidney specimen demonstrating kidney renal clear cell carcinoma<br>• microscopic view of kidney specimen demonstrating clear cell carcinoma<br>• microscopic view of kidney specimen demonstrating renal clear cell carcinoma<br>• microscopic view of kidney specimen demonstrating renal cell carcinoma clear cell type<br>• microscopic view of kidney specimen demonstrating RCC<br>• microscopic view of kidney specimen demonstrating kidney cancer |
| | 6 | • microscopic view of liver specimen demonstrating liver hepatocellular carcinoma<br>• microscopic view of liver specimen demonstrating liver cancer<br>• microscopic view of liver specimen demonstrating liver carcinoma<br>• microscopic view of liver specimen demonstrating hepatocellular carcinoma |
| | 7 | • microscopic view of lung specimen demonstrating lung adenocarcinoma<br>• microscopic view of lung specimen demonstrating NSCLC adenocarcinoma<br>• microscopic view of lung specimen demonstrating lung adenocarcinoma |

(Continued on next page)

(Continued)

| Condition | Class | Prompt |
|---|---|---|
| | 8 | • microscopic view of lung specimen demonstrating lung squamous cell carcinoma<br>• microscopic view of lung specimen demonstrating NSCLC squamous carcinoma<br>• microscopic view of lung specimen demonstrating squamous cell lung cancer |
| | 9 | • microscopic view of ovary specimen demonstrating ovarian cancer<br>• microscopic view of ovary specimen demonstrating ovarian serous cancer<br>• microscopic view of ovary specimen demonstrating serous carcinoma<br>• microscopic view of ovary specimen demonstrating ovarian serous cystadenocarcinoma |
| | 10 | • microscopic view of stomach specimen demonstrating stomach adenocarcinoma<br>• microscopic view of stomach specimen demonstrating stomach cancer<br>• microscopic view of stomach specimen demonstrating gastric cancer<br>• microscopic view of stomach specimen demonstrating gastric carcinoma |
| Tissue Types | | |
| | 1 | • HE histopathology image from the appendix<br>• microscopic region of a cross-section of the appendix<br>• microscopic view of the appendix<br>• portion of the appendix on histopathology<br>• HE-stained image of the appendix showing the tubular structure |
| | 2 | • HE histopathology image of breast tissue<br>• microscopic view of breast tissue<br>• region of breast tissue on histopathology<br>• HE section of breast tissue |
| | 3 | • HE histopathology image of the cervix<br>• microscopic view of the cervix demonstrating epithelial cells<br>• cervical tissue on histopathology<br>• region of HE-stained section of the cervix |

(Continued on next page)

(Continued)

| Condition | Class | Prompt |
|---|---|---|
| | 4 | • HE histopathology image of the colon<br>• colonic mucosa region on histopathology<br>• microscopic view of the colon<br>• HE-stained image highlighting portion of colon tissue<br>• colon tissue on histopathology |
| | 5 | • HE histopathology image of the fallopian tube<br>• microscopic cross-section of the fallopian tube<br>• microscopic view of the fallopian tube<br>• HE-stained section of the fallopian tube<br>• fallopian tube tissue on histopathology |
| | 6 | • HE histopathology image of the gallbladder<br>• microscopic view of the gallbladder<br>• HE-stained section showing a region of the gallbladder<br>• gallbladder tissue on histopathology |
| | 7 | • HE histopathology image of the liver<br>• microscopic view of liver tissue demonstrating hepatocytes arranged in cords<br>• liver tissue on histopathology |
| | 8 | • HE histopathology image of a lymph node<br>• microscopic view of a lymph node<br>• lymph node demonstrating mixture of immune cells predominantly lymphocytes<br>• HE-stained section of a lymph node<br>• lymph node tissue on histopathology |
| | 9 | • HE histopathology image of the ovary<br>• microscopic view of the ovary<br>• microscopic region of ovarian tissue<br>• HE-stained section of the ovary<br>• ovarian tissue on histopathology |
| | 10 | • HE histopathology image of the placenta<br>• placental tissue showcasing chorionic villi<br>• microscopic view of the placenta<br>• HE-stained section of the placenta<br>• placental tissue on histopathology |
| | 11 | • HE histopathology image of the prostate<br>• microscopic view of prostate tissue<br>• small region of prostate tissue with acini and stroma<br>• HE-stained section of the prostate<br>• prostate tissue on histopathology |

(Continued on next page)

(Continued)

| Condition | Class | Prompt |
|---|---|---|
| | 12 | • HE histopathology image of the skin<br>• microscopic view of the skin<br>• HE-stained section of the skin<br>• skin tissue on histopathology |
| | 13 | • HE histopathology image of the thyroid<br>• microscopic view of the thyroid<br>• HE-stained section of the thyroid<br>• thyroid tissue on histopathology |
| | 14 | • HE histopathology image of the upper GI tract such as the stomach<br>• upper GI mucosa region on histopathology<br>• microscopic view of the upper GI tract<br>• HE-stained image highlighting portion of upper GI tract tissue<br>• upper GI tract tissue on histopathology<br>• stomach or small intestine tissue on histopathology |
| | 15 | • HE histopathology image of the uterus<br>• microscopic view of the cells of the uterus<br>• HE-stained section of the uterus<br>• uterine tissue on histopathology |
| | 16 | • HE histopathology image of the vas deferens<br>• microscopic view of the vas deferens<br>• HE-stained section of the vas deferens<br>• vas deferens tissue on histopathology |

**Supplementary Table 15 | Zero shot prompts for MedGemma text-based medical evaluations.**

| Dataset | Prompt |
|---|---|
| MedQA | "Instructions: The following are multiple choice questions about medical knowledge. Solve them in a step-by-step fashion, starting by summarizing the available information. Output a single option from the four options as the final answer. Question: " + `<QUESTION>` + " Response (think step by step and then end with "Final Answer:" followed by *only* the letter corresponding to the correct answer enclosed in parentheses)" |
| PubMedQA | "Instructions: The following are multiple choice questions about medical knowledge. Solve them in a step-by-step fashion, starting by summarizing the available information. Output a single option from the four options as the final answer. Answer the following question given the context (reply with one of the options): Context: " + `<CONTEXT>` " + Question: " + `<QUESTION>` + " Response (think step by step and then end with "Final Answer:" followed by *only* the letter corresponding to the correct answer enclosed in parentheses)" |
| MedMCQA | "Instructions: The following are multiple choice questions about medical knowledge. Solve them in a step-by-step fashion, starting by summarizing the available information. Output a single option from the four options as the final answer. Question: " + `<QUESTION>` + "Response (think step by step and then end with "Final Answer:" followed by *only* the letter corresponding to the correct answer enclosed in parentheses)" |
| MMLU | "Instructions: The following are multiple choice questions about medical knowledge. Solve them in a step-by-step fashion, starting by summarizing the available information. Output a single option from the four options as the final answer. Question: " + `<QUESTION>` + " Response (think step by step and then end with "Final Answer:" followed by *only* the letter corresponding to the correct answer enclosed in parentheses)" |
| MedXpertQA | `<QUESTION>` + " Response (think step by step through each of the multiple choice options. You MUST end your response with 'Final Answer:' followed by *only* the letter corresponding to the correct answer enclosed in parentheses) like 'Final Answer:(X)'." |

**Supplementary Table 16 | Zero-shot prompts for MedGemma image classification and VQA evaluations.** For all but the medical MedGemma and Med-Gemini models, the prompts below were prefixed with the following system message (SM): MI: "You are a helpful medical assistant.", or RI: "You are a helpful radiology assistant.", as indicated.

| Dataset(s) | SM | Prompt/Example |
|---|---|---|
| MIMIC-CXR, CheXpert | MI | <IMAGE> + " Is there <CONDITION> in this image? You may write out your argument before stating your final very short, definitive, and concise answer (if possible, a single word or the letter corresponding to your answer choice) X in the format "Final Answer: X":"<br><br>Where <CONDITION> is one of the following:<br>• For MIMIC-CXR & CheXpert: atelectasis, cardiomegaly, consolidation, edema, pleural effusion.<br>• For CXR14: lung opacity, pneumothorax.<br>*Exception for CXR14*: The fracture condition uses the question "Are there any fractures in the image?". |
| PathMCQA | MI | "<QUESTION>"<br><br>Example for <QUESTION>:<br>"What is the predominant diagnostic finding in this cervical biopsy image? Please select the correct option from the following choices: A) Normal or benign tissue B) Mild dysplasia (CIN1) C) Moderate or severe dysplasia (CIN 2+)" |
| US-Derm MCQA | MI | <IMAGE> + "<QUESTION> You may write out your argument before deciding on the most likely condition X (with X being one of: A, B, C, or D) in the format: "The most likely diagnosis is: X":"<br><br>Example for <QUESTION>:<br>"Question: Given the following image, what is the most likely dermatology condition? Options: (A) Granuloma annulare. (B) Melanocytic Nevus. (C) Erythema annulare centrifugum. (D) Morphea/Scleroderma." |
| EyePACS | MI | <IMAGE> + "Given this fundus image, determine the most likely diabetic retinopathy (DR) stage present, even if you are unsure:<br>A: No DR<br>B: mild DR<br>C: moderate DR<br>D: severe DR<br>E: proliferative DR<br>You may write out your argument before stating your final, very short, definitive, and concise answer (no more than a few words) and letter corresponding to your answer choice X in the format "The most likely diagnosis is: X":" |
| SLAKE | MI | <IMAGE> + <QUESTION> + "You may write out your argument before stating your final very short, definitive, and concise answer (if possible, a single word or the letter corresponding to your answer choice) X in the format "Final Answer: X": " |
| VQA-RAD | RI | <IMAGE> + "Given this radiology image, which can be a frontal chest X-ray, a single slice head or abdominal CT or MR image, provide a very short, definitive, and concise answer (if possible, a single word) to the following question: <QUESTION>" |
| MedXpertQA (multi-modal questions) | MI | "Figure A" + <IMAGE_A> + "Figure B" + <IMAGE_B> + ... + "<QUESTION> Think step by step through each of the multiple choice options. You MUST end your response with 'Final Answer:' followed by only the letter corresponding to the correct answer enclosed in parentheses)." |
| CXR report generation | - | <IMAGE> "<INDICATION> findings:"<br><br>Where <INDICATION> is the indication section of the report as prefix. |

## H. Comparison of CXR data-efficient learning

To compare with previous research on CXR image encoder-based data-efficient learning, we compared MedSigLIP to ELIXR [24]. Performance of MedSigLIP and ELIXR are compared for various CXR findings at different training set sizes on CheXpert (Supplementary Fig. 3) and CXR14 (Supplementary Fig. 4).

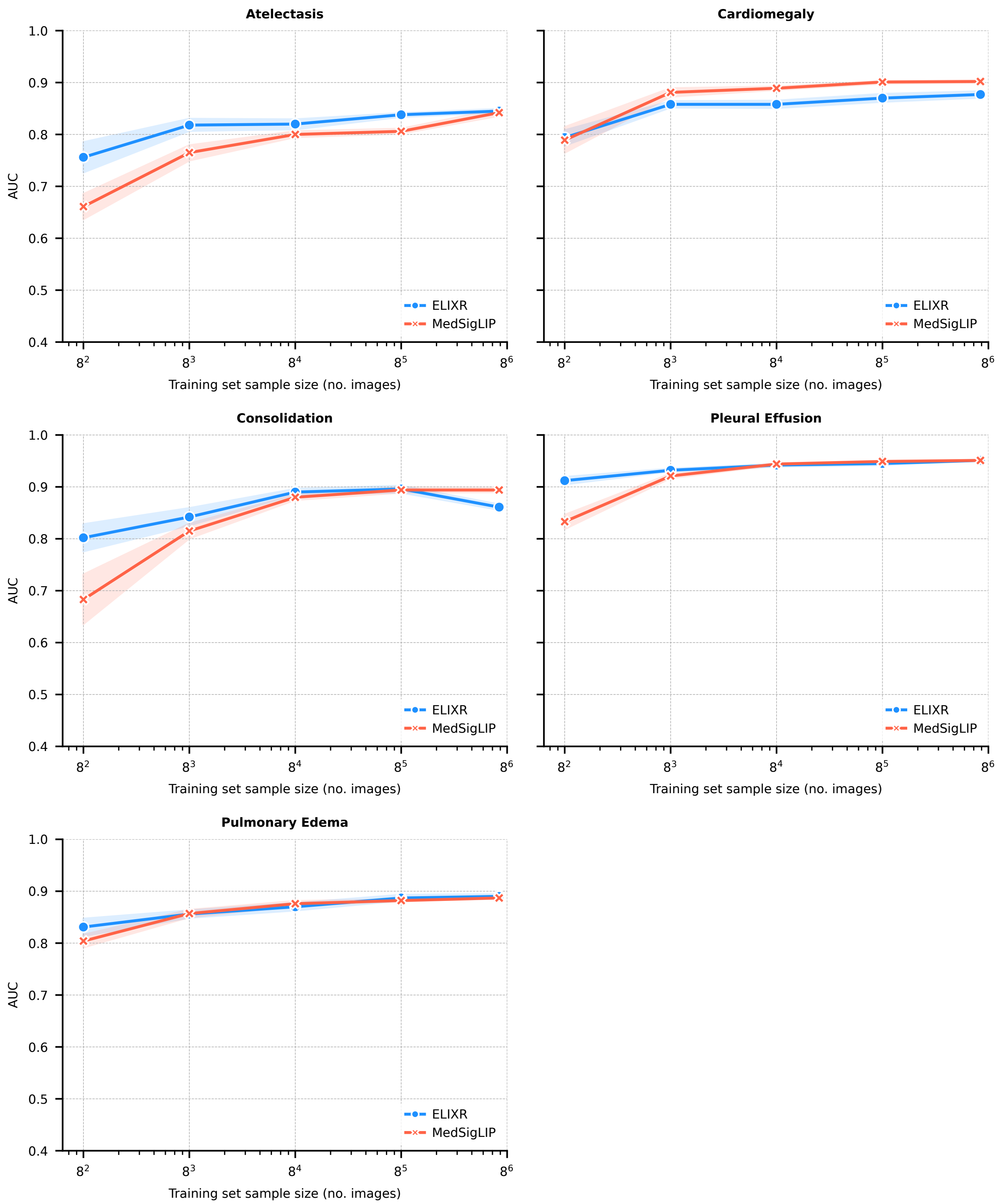


**Supplementary Figure 3 | Condition-based results for MedSigLIP CXR data efficient learning on CheXpert datasets comparing to ELIXR [24].** Shading represents 95% confidence intervals which were derived via non-parametric bootstrap resampling with 1000 iterations across independent test samples; no technical replicates or repeated measurements were used.

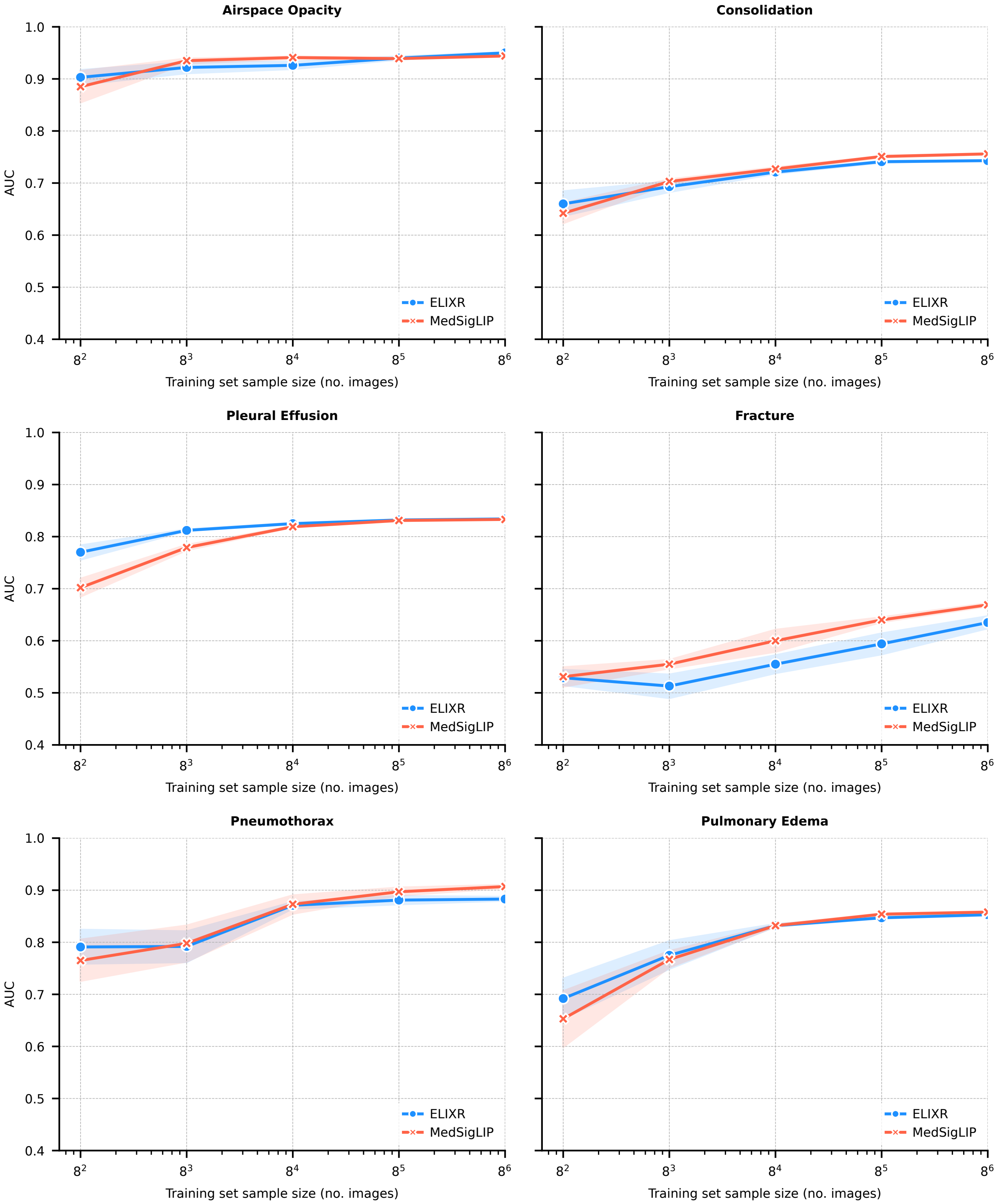


**Supplementary Figure 4 | Condition-based results for MedSigLIP CXR data efficient learning on CXR14 datasets comparing to ELIXR [24].** Shading represents 95% confidence intervals which were derived via non-parametric bootstrap resampling with 1000 iterations across independent test samples; no technical replicates or repeated measurements were used.

# I. Additional evaluation using an internal synthetic EHR dataset: EHRQA

## I.1. Fine-tuned performance for EHRQA

MedGemma 27B Text did not initially include EHR data or examples in training and demonstrated a performance gap relative to larger models. To further evaluate and demonstrate potential to improve performance of MedGemma for a specific task of interest, we fine-tuned MedGemma 27B Text using RL on the EHRQA training set described below. This task-adaptation resulted in substantial improvement as shown in Supplementary Table 17. Detailed results are shown in Supplementary Fig. 5, which indicates that the greatest gains were observed in question categories requiring reasoning across inter-dependent records. This experiment underscores the potential of fine-tuning MedGemma for effective use in task-specific EHR applications.

**Supplementary Table 17 | EHRQA accuracy results before and after fine-tuning via RL.** Bold indicates the best result for small models.

| | Small Models | | | Large Models | | |
|---|---|---|---|---|---|---|
| **Type** | **MedGemma 27B Text** | **Gemma 3 27B** | **MedGemma 27B Text (RL)** | **Gemini 2.5 Flash** | **Gemini 2.5 Pro** | **o3** |
| **EHRQA** (n=4377) | 86.3 | 84.2 | **93.6** | 95.0 | 95.4 | 92.5 |

## I.2. EHRQA additional information

While numerous benchmarks assess the ability of language models to reason over EHRs, including emrQA [35], emrKBQA [36], EHRSQL [37], EHRNoteQA [38], and MedAlign [39], we developed a new evaluation for MedGemma specifically focused on longitudinal, outpatient EHR data. Our evaluation, which we call EHRQA, is based on a programmatic framework to generate question-answer (QA) pairs from synthetic, text and FHIR-formatted records. These records were produced using Synthea [40], a tool that simulates complete patient medical histories based on population statistics and disease progression models. From an initial 100 synthetic records, we selected 81 that fit within a 32k context window, splitting them into training (42), validation (20), and test (19) sets. Each patient record is comprehensive, containing hundreds to thousands of FHIR entries across various resource types like Conditions, Medications, and Observations (and a corresponding plain-text representation). A key limitation of this dataset, however, is the absence of clinical notes, which we hope to address in future work.

Our QA generation framework treated each patient's synthetic record as a collection of ground truth facts. It expanded upon these facts through a multi-hop reasoning process [41] that integrated information from external medical knowledge bases (SNOMED, RxNorm, LOINC), applied programmatic reasoning (e.g., temporal, arithmetic), and leveraged inter-dependencies within the patient's data programmatically. These derived facts are then converted into natural language QA pairs using templated questions and large language model (LLM) rephrasing. In collaboration with clinicians, we developed 42 distinct question types, grouped into 10 categories, to reflect queries from both healthcare professionals and consumers. This process generated approximately 200 questions per patient. In total, there are 10,437 QA examples in the training split, 5,133 QA examples in the validation split, and 4,377 QA examples in the test split. All questions were designed for automated evaluation (e.g., regex matching) and support multiple-choice, Yes/No, and simple free-response formats. For evaluation, the model is prompted with a condensed, plain-text representation of the patient's FHIR data and the corresponding question. Final accuracies are calculated by averaging the scores across the 10 question categories. Details of EHRQA question categories are shown in Supplementary Table 18 and prompts for question rewrites and evaluation are shown in Supplementary Table 19.

**Supplementary Table 18 | EHRQA question categories with original (template-generated) and LLM-rephrased examples.**

| Category | Description | Example: templated | Example: LLM rephrased |
|---|---|---|---|
| Condition existence (transitive) | Questions related to whether a patient has a history of a general class of medical conditions. | Has the patient had a medical condition in gingival structure in the past? | Has the patient had a past medical condition affecting the gums? |
| Condition existence | Questions related to whether a patient has a history of a given medical condition. | Has the patient had acute bronchitis in the past? | Has the patient had acute bronchitis previously? |
| Immunization date | Questions related to the existence of patient immunizations and their immunization dates. | When did the patient last receive Hep B, adult immunization? | When was the patient's last adult hepatitis B vaccination administered? |
| Medication current | Questions related to whether the patient has active prescriptions for a given medication. | Does the patient have a prescription for {28 (norethindrone 0.35 MG Oral Tablet) } Pack? | Does the patient have an active prescription for norethindrone oral tablet? |
| Medication existence | Questions related to whether the patient has ever taken a given medication or type of medication. | Did the patient have a prescription for insulin isophane, human 70 UNT/ML / insulin, regular, human 30 UNT/ML Injectable Suspension in the past? | Did the patient have a prescription for human insulin isophane / human insulin regular injectable suspension in the past? |
| Medication prescriber | Questions related to the prescriber of a given medication for a patient. | Who prescribed acetaminophen 325 MG Oral Tablet on the date 2022-06-30? | Who prescribed acetaminophen on June 30, 2022? |
| Medication prescription date | Questions related to the medication prescription dates. | When was acetaminophen 325 MG Oral Tablet first prescribed? | When was acetaminophen oral tablet first prescribed? |
| Medication for condition | Questions on the relationships between medication and medical condition history. | Which condition were the medications acetaminophen 300 MG / hydrocodone bitartrate 5 MG Oral Tablet prescribed for? | For what condition was acetaminophen/hydrocodone prescribed? |
| Observation (dependent) | Questions related to patient lab observations related to some medical event. | What was the patient's first recorded value for Pain severity - 0-10 verbal numeric rating [Score] - Reported after they were first prescribed Acetaminophen 325 MG Oral Tablet? | What was the patient's initial reported pain score after the first prescription of acetaminophen 325 mg oral tablet? |
| Observation value | Questions related to patient lab observations and their values. | What is the patient's most recent recorded value for Heart rate? | What is the patient's most recent heart rate reading? |

**Supplementary Table 19 | Prompts for EHRQA construction and evaluation.**

| Purpose | Prompt |
|---|---|
| **General question rewrite** | You are a helpful medical editing assistant.<br>You will be presented with a question which pertains to the medical record for a patient. Rewrite the question in a way that sounds more natural, omitting unnecessary (in particular parenthesized) details but retaining enough detail to leave the question unambiguous.<br>- Use correct capitalization and language appropriate for a medical professional.<br>- Avoid contractions in the question, but use common-language terminology where that is possible without causing ambiguity.<br>- Keep in mind that the question may be a multiple choice question followed by several answer choices.<br>- Be sure the rewritten questions ask about the same topic as the original. For instance, if there is a question about who prescribed a medication, the re-written question should do the same.<br>ORIGINAL QUESTION: `<ORIGINAL_QUESTION>`<br>REWRITTEN QUESTION: |
| **Medication question rewrite** | You are a helpful medical editing assistant.<br>You will be presented with a question which pertains to the medical record for a patient. Rewrite the question in a way that sounds more natural, omitting unnecessary (in particular parenthesized) details but retaining enough detail to leave the question unambiguous.<br>- Use correct capitalization and language appropriate for a medical professional.<br>- Avoid contractions in the question, but use common-language terminology where that is possible without causing ambiguity.<br>- Keep in mind that the question may be a multiple choice question followed by several answer choices.<br>- If the brand name is mentioned in the original question, rewrite the question using that brand name only.<br>- Remove the medication strength/dosage information in the medication name.<br>- Use common names for medication classes if available.<br>EXAMPLES: `<FEW_SHOT_EXAMPLES>`<br>ORIGINAL QUESTION: `<ORIGINAL_QUESTION>`<br>REWRITTEN QUESTION: |
| **Evaluation** | You are a medical assistant specializing in answer questions about a patient electronic health records (EHR). You will be provided with a patient's full EHR data and a question for you to answer.<br>======= Patient EHR Data =======<br>`<PATIENT_CONTEXT>`<br>======= End Patient EHR Data =======<br>Based on the data provided in the patient's EHR, please answer concisely with no explanation. If the question is a multiple choice question, answer with only the letter of the correct choice, such as "B". If the question is a free response question, answer with the correct data. In either case, remember to not provide an explanation.<br>Question: `<QUESTION>`<br>Answer choices: `ANSWER_CHOICES` |

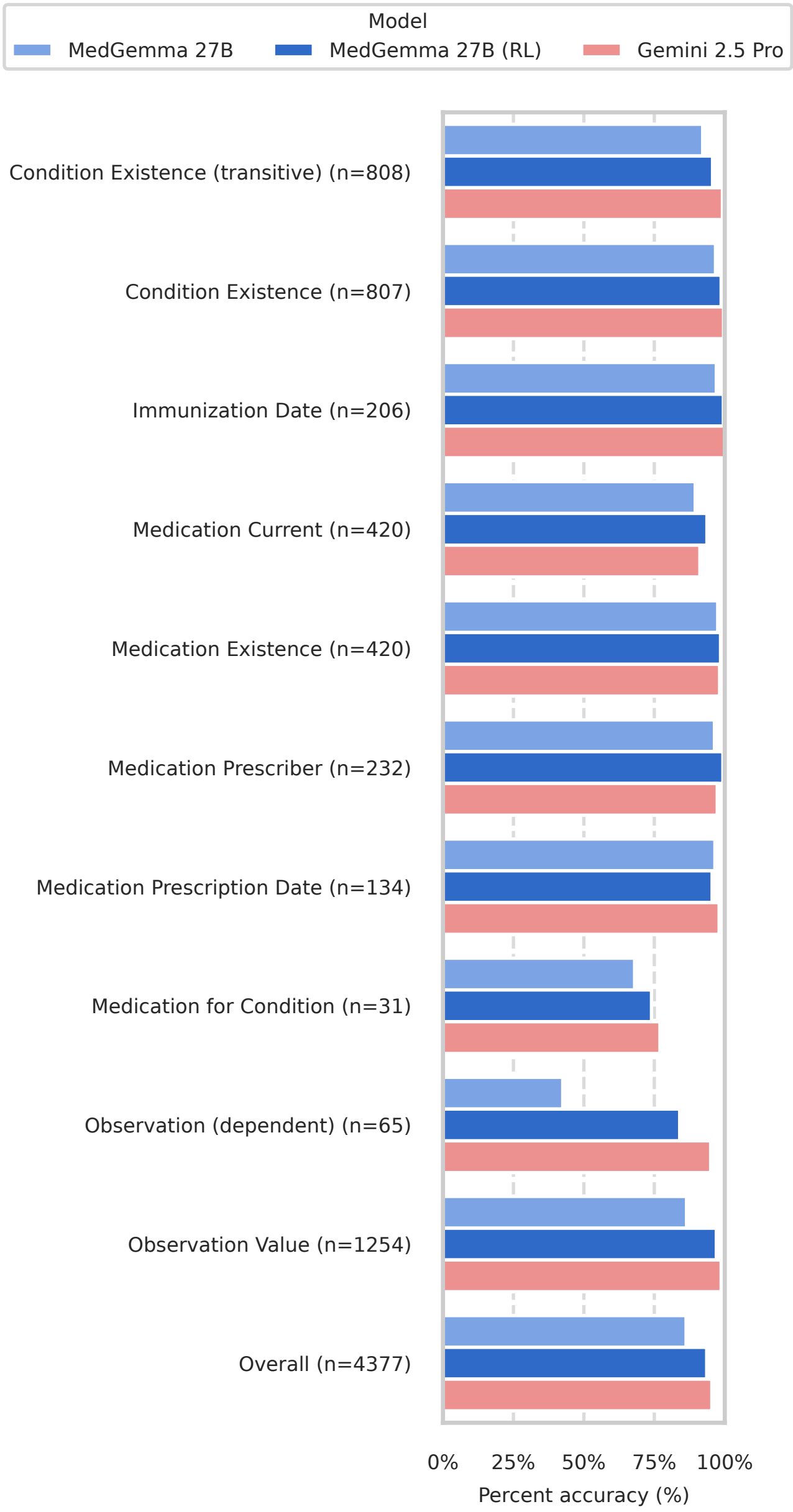


**Supplementary Figure 5 | EHR question answering results by question type.** Accuracy results for MedGemma 27B Text on EHRQA, before and after RL-tuning, and compared to Gemini 2.5 Pro across different subcategories of question types from the EHRQA dataset.

## J. Medical reasoning examples

Examples of MedGemma 27B Text medical reasoning output based on clinical vignettes are provided for oncology in Supplementary Table 20 and Supplementary Table 21, for cardiology in Supplementary Table 22 and Supplementary Table 23, and for infectious diseases in Supplementary Table 24 and Supplementary Table 25. Clinical commentary on the MedGemma output is also provided for reference.

**Supplementary Table 20 | Example of MedGemma 27B Text clinical reasoning in an oncology setting.** Commentary is provided by a US board-certified practicing oncologist.

**Prompt**

A 62-year old man presents with a 2 cm spiculated right upper lobe peripheral lung nodule and mediastinal lymphadenopathy. Imaging furthermore shows multiple metastatic lesions in the liver and bones, and a solitary 1 cm lesion in the right parietal lobe without surrounding vasoedema. A biopsy from the liver demonstrated adenocarcinoma consistent with a lung primary. Genomic sequencing demonstrated TP53 and EGFR L858R mutation, and PD-L1 was 70%. On examination, the patient is without any neurologic deficits and is ECOG 0. What is the best next step in management for this patient?

**MedGemma 27B Text output**

Okay, let's break down the management for this patient with Stage IV NSCLC.
**Key Findings:**
1. **Diagnosis:** Stage IV Adenocarcinoma of the Lung (confirmed by liver biopsy).
2. **Driver Mutation:** EGFR L858R mutation (highly actionable).
3. **Biomarker:** PD-L1 70% (high expression).
4. **Metastases:** Widespread (mediastinal nodes, liver, bone, solitary brain lesion).
5. **Clinical Status:** Excellent performance status (ECOG 0), asymptomatic brain lesion.

**Analysis & Rationale:**
- **EGFR Mutation is Paramount:** The presence of the EGFR L858R mutation is the most critical factor guiding first-line therapy. Patients with EGFR-mutated NSCLC have demonstrated significantly improved outcomes with EGFR tyrosine kinase inhibitors (TKIs) compared to chemotherapy.
- **PD-L1 High:** While PD-L1 is high (70%), indicating potential benefit from immunotherapy, current guidelines and clinical trial data (like FLAURA) strongly favor EGFR TKIs as the *first-line* treatment for patients with EGFR mutations, even those with high PD-L1. Immunotherapy is typically considered after TKI failure or in combination with chemotherapy in specific settings, but not usually as the initial monotherapy in this scenario.
- **Brain Metastasis:** The solitary, asymptomatic brain lesion needs management. EGFR TKIs, particularly third-generation agents like Osimertinib, have good central nervous system (CNS) penetration and activity. Therefore, systemic therapy with an EGFR TKI is often the initial approach to treat the brain metastasis as well. Close monitoring with MRI is essential. If the lesion progresses despite systemic therapy or becomes symptomatic, Stereotactic Radiosurgery (SRS) would be considered.
- **ECOG 0:** The patient's excellent performance status makes him a suitable candidate for aggressive systemic therapy.

**Best Next Step in Management:** The best next step is to initiate **first-line systemic therapy with an EGFR tyrosine kinase inhibitor (TKI)**.
- **Preferred Agent: Osimertinib** is generally the preferred first-line EGFR TKI due to its superior efficacy (progression-free survival and overall survival) and improved CNS penetration compared to earlier-generation TKIs (like Gefitinib, Erlotinib, Afatinib) demonstrated in the FLAURA trial.
- **Management of Brain Metastasis:** Start Osimertinib. Monitor the brain lesion closely with serial MRI scans (e.g., every 3-6 months initially). Consider SRS if the lesion progresses or causes symptoms.
- **Supportive Care:** Address potential side effects of the TKI (e.g., rash, diarrhea). Manage potential bone pain with analgesics or palliative radiation if needed.

**Commentary by US board-certified practicing oncologist**

**Strengths:** Identifies the key components of staging and the actionable driver mutation. PD-L1 is high but correctly identifies TKI as preferable over immunotherapy. Correctly identifies osimertinib as having good brain penetration especially with asymptomatic and small brain lesions. Appropriately highlights FLAURA trial. Includes supportive care aspects including potential side effects.
**Areas for Improvement:** Immunotherapy can be considered after TKI progression, though the treatment paradigm has evolved (i.e., amivantamab + chemotherapy, datopotamab deruxtecan). Although osimertinib is a correct answer, newer combinations are available and have shown benefit over osimertinib alone (i.e., amivantamab + lazertinib, osimertinib + chemotherapy). Serial brain MRI is mentioned but serial rest-of-body imaging is not. Dosing of osimertinib not mentioned. Bone disease discussed, though no consideration for bone modifying agents or for whether surgery may be indicated. Shared decision-making would be a beneficial addition.

**Supplementary Table 21 | Example of MedGemma 27B Text clinical reasoning in an oncology setting, specifically prompted for *succinct* output.** Commentary is provided by a US board-certified practicing oncologist.

| **Prompt** |
|---|
| *Same as in Supplementary Table 20 with the suffix: Please be succinct.* |
| **MedGemma 27B Text output** |
| Initiate Osimertinib and monitor the brain lesion with serial MRI. |
| **Commentary by US board-certified practicing oncologist** |
| **Strengths:** To the point, directly addresses systemic therapy + serial brain MRI.<br>**Areas for Improvement:** A little too succinct, does not discuss radiosurgery, and otherwise the same issues as the lengthy output in the prior example. |

**Supplementary Table 22 | Example of MedGemma 27B Text clinical reasoning in a cardiology setting.** Commentary is provided by a US board-certified practicing cardiologist.

| **Prompt** |
|---|
| A 55-year old woman with active smoking history, type II diabetes, hypertension, history of coronary artery disease was recently hospitalized for heart failure with an ejection fraction of 35%. She is presenting for follow-up post discharge. Her discharge medications included lisinopril 10 mg daily, carvedilol 2.5 mg twice daily, and eplerenone 25 mg daily, in addition to her usual medications for diabetes. In clinic, she reports feeling symptomatic with dyspnea after going up 1 flight of stairs though is asymptomatic at rest.<br>Vital signs at rest: BP 130/90, HR 75, RR 10, Temperature 97.2F, SpO2 98% on room air. Labs are remarkable for Na 132 mEq/L, K 4.9 mEq/L, BUN 22 mg/dL, Cr 1.04 mg/dL.<br>The patient reports having a friend on Entresto. Please review whether Entresto is indicated in this patient and why or why not. |

*Continued on next page*

**MedGemma 27B Text output**

Okay, let's review the indication for Entresto (sacubitril/valsartan) in this patient.
**Patient Summary:**

- **Diagnosis:** Heart Failure with Reduced Ejection Fraction (HFrEF), EF 35%.
- **Symptoms:** Dyspnea on exertion (1 flight of stairs), NYHA Class II-III.
- **Current HFrEF Medications:** Lisinopril (ACE inhibitor), Carvedilol (Beta-blocker), Eplerenone (MRA).
- **Comorbidities:** Smoking, Type II Diabetes, Hypertension, CAD.
- **Relevant Labs:** K 4.9 mEq/L (high-normal), Cr 1.04 mg/dL (mildly elevated/high-normal).
- **Vitals:** BP 130/90 mmHg (acceptable for initiation).

**Entresto Indication Review:**
Entresto (sacubitril/valsartan) is indicated for the reduction of cardiovascular death and hospitalization for heart failure in adult patients with chronic heart failure (NYHA class II-IV) with reduced ejection fraction (LVEF ≤ 40%).
**Analysis for this Patient:**

1. **Ejection Fraction:** The patient's EF is 35%, which meets the criteria (≤ 40%).
2. **Symptom Status:** The patient is symptomatic (dyspnea on exertion) despite being on foundational guideline-directed medical therapy (GDMT) including an ACE inhibitor (lisinopril), a beta-blocker (carvedilol), and an MRA (eplerenone). This is the target population for Entresto.
3. **Contraindications:**
   - **ACE Inhibitor Use:** Entresto should *not* be used concurrently with an ACE inhibitor (like lisinopril) due to increased risk of angioedema, hypotension, and hyperkalemia. The lisinopril would need to be stopped, typically with a 36-hour washout period, before starting Entresto.
   - **Angioedema History:** No history of angioedema related to ACE inhibitors or ARBs is mentioned.
   - **Hypotension:** Her current BP of 130/90 mmHg is generally acceptable for initiating Entresto, although close monitoring is needed.
   - **Renal Function:** Her creatinine is 1.04 mg/dL. While Entresto can cause a small increase in creatinine, this level is not a contraindication, but renal function should be monitored.
   - **Potassium:** Her potassium is 4.9 mEq/L (high-normal). This requires close monitoring, as Entresto can increase potassium levels, especially in combination with an MRA like eplerenone.

**Conclusion:**
**Yes, Entresto is indicated for this patient.**
**Reasoning:** She has symptomatic HFrEF (EF 35%) despite being on appropriate foundational therapy (ACE inhibitor, beta-blocker, MRA). Clinical trials have demonstrated that switching from an ACE inhibitor (like lisinopril) to Entresto significantly reduces morbidity and mortality in this patient population.
**Important Considerations:**

- **Lisinopril Discontinuation:** The lisinopril must be stopped before starting Entresto.
- **Monitoring:** Close monitoring of blood pressure, heart rate, renal function (creatinine/eGFR), and potassium levels is crucial, especially after initiation and dose titration.
- **Dosing:** Entresto is typically started at a low dose (e.g., 24/25 mg BID) and titrated upwards to the target dose (e.g., 49/51 mg BID or 97/103 mg BID) as tolerated, based on guideline recommendations.

This represents a significant therapeutic opportunity to improve her heart failure outcomes.

**Commentary by a US board-certified practicing cardiologist**

**Strengths:** Correctly states that entresto is indicated for the reduction of CV death and HF hospitalization. Appropriately checks renal function, electrolytes, angioedema and BP. Lists washout and monitoring.
**Areas for Improvement:** Dose is incorrect for the lowest dose of entresto. Minor nit that entresto has now been shown in meta-analysis to reduce all cause mortality in EF<40% (not just cardiac mortality as stated) [42]. Creatinine is mentioned but estimated GFR is the more useful measure. Minor but no mention of carvedilol dose. The dose is very low and should be increased. If it is increased, there will be less BP room for entresto so would need to titrate both carefully but can be done rapidly (Strong HF trial). The other thing that could have been mentioned is empaglaflozin which is not included in this list. But the question is specific to entresto so understandable.

**Supplementary Table 23 | Example of MedGemma 27B Text clinical reasoning in a cardiology setting with *succinct* output.** Commentary is provided by a US board-certified practicing cardiologist.

| **Prompt** |
|---|
| *Same as in Supplementary Table 22 with the suffix: Please be succinct.* |
| **MedGemma 27B Text output** |
| **Yes, Entresto is indicated.**<br>**Rationale:** This patient has symptomatic Heart Failure with reduced Ejection Fraction (HFrEF) (EF 35%, NYHA Class II) despite being on foundational guideline-directed medical therapy (ACE inhibitor, beta-blocker, MRA). Entresto (sacubitril/valsartan) is recommended to replace the ACE inhibitor (lisinopril) in such patients to further reduce cardiovascular death and heart failure hospitalizations. Her current BP and potassium levels are acceptable for initiation, although close monitoring is required. |
| **Clinical commentary by a US board-certified practicing cardiologist** |
| **Strengths:** A more useful answer - short and to the point, including the rationale for the treatment.<br>**Areas for Improvement:** Does not include renal function in the list of things to monitor (just BP and potassium). Same note as above that entresto has now been shown in meta-analysis to reduce all cause mortality in EF<40% rather than just cardiac mortality as stated. |

**Supplementary Table 24 | Example of MedGemma 27B Text clinical reasoning in an infectious disease setting.** Commentary is provided by a UK Specialist Registrar in infectious diseases.

| **Prompt** |
|---|
| A 17-year old otherwise healthy man presents in the emergency room for fatigue and spiking fevers. Two weeks prior, he reports having felt flu-like symptoms with fever, nausea, vomiting, and cough. Symptoms persisted but have worsened, with new onset shortness of breath, headache, and abdominal discomfort. There have been no sick contacts at home. He has been backpacking across various regions in the United States, most recently in Hawaii.<br>On evaluation, vital signs are remarkable for SBP 115/85, HR 110, RR 14, Temperature 102.2F, SpO2 90% on room air. Exam is remarkable for bilateral crackles and icterus.<br>Labs show the following:<br>• WBC: 18.2 × $10^3$/mcL, Hb: 15.5 g/dL, Plt: 120 × $10^3$/mcL<br>• Na: 142 mEq/L, K: 5.1 mEq/L, Cl: 101 mEq/L, $CO_2$: 26 mEq/L, BUN: 46 mg/dL, Cr: 2.2 mg/dL<br>• ALP: 84 U/L, ALT: 175 U/L, AST: 140 U/L, Total bilirubin: 2.0 mg/dL<br>CXR shows bilateral pulmonary infiltrates. A follow-up echocardiogram shows EF 40%. A respiratory viral panel was negative. Blood, sputum, and urine cultures are taken.<br>What would be the next best steps in terms of diagnostic testing to establish the etiology of his symptoms? |

*Continued on next page*

**MedGemma 27B Text output**

**Rationale Breakdown:**

1. **Clinical Presentation Analysis:** The patient presents with a severe, multi-system illness characterized by fever, fatigue, shortness of breath, headache, abdominal discomfort, jaundice (icterus), hypoxia, leukocytosis, thrombocytopenia, acute kidney injury (elevated BUN/Cr), hepatitis (elevated ALT/AST/bilirubin), and possible myocarditis (reduced EF on echo). The illness began with flu-like symptoms two weeks ago and has progressively worsened.
2. **Key Clues:**
   - **Travel History:** Recent travel to Hawaii is a significant clue, pointing towards infections endemic to that region.
   - **Multi-organ Involvement:** The combination of pulmonary infiltrates, jaundice, AKI, and myocarditis suggests a systemic process, often infectious.
   - **Specific Lab Findings:** Leukocytosis with thrombocytopenia, elevated liver enzymes, and bilirubin are important diagnostic pointers.
3. **Differential Diagnosis (Focusing on Hawaii-Endemic Infections):**
   - **Leptospirosis:** Highly consistent with the presentation. Causes fever, headache, myalgia, conjunctival suffusion (not mentioned but common), jaundice, renal failure, pulmonary hemorrhage/ARDS, and myocarditis. Thrombocytopenia is common; leukocytosis can occur. Hawaii is an endemic area.
   - **Dengue Fever:** Endemic in Hawaii. Causes fever, headache, myalgia, rash (not mentioned), leukopenia (patient has leukocytosis), thrombocytopenia. Severe dengue can cause organ impairment, but the specific combination of jaundice, AKI, and myocarditis with leukocytosis makes it less likely than leptospirosis.
   - **Hantavirus Pulmonary Syndrome (HPS):** Endemic in Hawaii. Causes fever, myalgia, headache, followed by respiratory failure and pulmonary edema. Thrombocytopenia and leukocytosis are typical. However, significant jaundice and myocarditis are less characteristic of HPS.
   - **Rickettsial Infections (e.g., Murine Typhus, Scrub Typhus):** Endemic in Hawaii. Can cause fever, headache, rash, myalgia, sometimes pneumonia, hepatitis, AKI.
   - **Other possibilities:** Influenza (severe complications), other viral hepatitis (EBV, CMV), bacterial sepsis, melioidosis (rare but reported).
4. **Prioritizing Diagnostic Tests:** Based on the clinical picture and travel history, Leptospirosis is the most likely diagnosis. Therefore, specific testing for Leptospirosis should be prioritized. Dengue and Hantavirus are also important considerations due to endemicity and overlapping features.
5. **Specific Diagnostic Tests:**
   - **Leptospirosis:**
     - **Serology:** ELISA for IgM antibodies is often the first step for faster results. Microscopic Agglutination Test (MAT) is the gold standard but takes longer. Paired sera (acute and convalescent) are often needed for definitive diagnosis.
     - **PCR:** Detects Leptospira DNA in blood (early) or urine (can be positive later, especially after the first week). Given the patient is 2 weeks into symptoms, urine PCR might be useful.
   - **Dengue:**
     - **Serology:** IgM/IgG antibodies.
     - **NS1 Antigen:** Detectable early in illness.
     - **PCR:** Detects viral RNA early in illness.
   - **Hantavirus:**
     - **Serology:** IgM/IgG antibodies.
     - **PCR:** Detects viral RNA early in illness.
   - **Other Tests:** While awaiting results for the primary suspects, continue monitoring organ function (liver, kidney), coagulation, and review pending cultures. Consider additional tests like EBV/CMV serology, HIV testing, and Rickettsial serology/PCR if the initial workup is negative or the clinical picture evolves.
6. **Conclusion:** The most crucial next steps are to order specific diagnostic tests for the most likely endemic infections given the patient's presentation and travel history, particularly Leptospirosis, Dengue, and Hantavirus.

**Final Answer:** The final answer is

| Order specific serological and PCR testing for Leptospirosis, Dengue, and Hantavirus |
|---|

*Continued on next page*

Supplementary Table 24 – *Continued from previous page*

| **Commentary by a UK Specialist Registrar in infectious diseases** |
|---|
| **Strengths:** Well-structured answer, making the information easy to parse. Correctly has the most likely diagnosis (lepto) as the focus of the answer. The suggested diagnostic testing enables confirmation of the specific diagnoses listed in the differential.<br>**Areas for Improvement:** Dengue fever, hantavirus and melioidosis don't seem to occur in Hawaii much, if at all (and at least, they are not endemic, as stated in the answer). The suggested diagnostic testing perhaps narrows towards specific diagnoses too quickly, whereas in reality broader testing may still be necessary (e.g. CSF analysis given headache, further chest imaging via CT, etc.) alongside more specific confirmatory tests. It could mention that many of these diagnostic tests are relatively unusual in most hospitals, often requiring samples to be sent via courier to specialized labs, and therefore there is a significant time lag associated with getting results. This may necessitate empiric treatment whilst awaiting for results, which is an important clinical consideration. |

**Supplementary Table 25** | **Example of MedGemma 27B Text clinical reasoning in an infectious disease setting with *succinct* output.** Commentary is provided by a UK Specialist Registrar in infectious diseases.

| **Prompt** |
|---|
| *Same as in Supplementary Table 24 with the suffix: Please be succinct.* |
| **MedGemma 27B Text output** |
| **Next best steps in diagnostic testing:**<br>1. Leptospirosis serology (IgM ELISA/MAT).<br>2. Hantavirus serology (IgM).<br>3. Dengue serology (IgM/IgG, NS1 antigen).<br>4. Blood and urine cultures.<br>5. Urinalysis.<br>6. Cardiac enzymes (Troponin).<br>7. Coagulation studies. |
| **Commentary by a UK Specialist Registrar in infectious diseases** |
| **Strengths:** Correctly lists confirmatory diagnostic tests for each suggested differential. Covers both specific confirmatory tests (to pinpoint a particular condition) and more generalized diagnostics (e.g. coagulation studies) that would yield more useful information to guide next steps in case the confirmatory diagnostic tests are negative.<br>**Areas for Improvement:** Same feedback as before regarding the epidemiology of hantavirus/dengue in Hawaii - seems to be rare, if at all present. Repeats several items already listed as being performed in the query (e.g. blood, urine cultures). Unclear why it limits the infectious disease testing to a single antibody (IgM) for each potential cause - it would be normal to perform IgM and IgG as both are useful in distinguishing between an acute infection versus a prior infection. No mention of molecular (genetic) testing which is increasingly used for diagnosis. |

## Supplementary references


1. DeepSeek-AI. *DeepSeek-R1: Incentivizing reasoning capability in LLMs via reinforcement learning* 2025. arXiv: 2501.12948 [cs.CL].
2. Muennighoff, N., Yang, Z., Shi, W., Li, X. L., Fei-Fei, L., Hajishirzi, H., Zettlemoyer, L., Liang, P., Candès, E. & Hashimoto, T. B. *s1: Simple test-time scaling* in *Proceedings of the 2025 Conference on Empirical Methods in Natural Language Processing* (2025), 20286–20332.
3. Singhal, K., Azizi, S., Tu, T., Mahdavi, S. S., Wei, J., Chung, H. W., Scales, N., Tanwani, A., Cole-Lewis, H., Pfohl, S., *et al.* Large language models encode clinical knowledge. *Nature* **620,** 172–180 (2023).

4. Johnson, A. E., Pollard, T. J., Berkowitz, S. J., Greenbaum, N. R., Lungren, M. P., Deng, C.-y., Mark, R. G. & Horng, S. MIMIC-CXR, a de-identified publicly available database of chest radiographs with free-text reports. *Scientific data* **6,** 317 (2019).
5. Johnson, A., Pollard, T., Mark, R., Berkowitz, S. & Horng, S. *MIMIC-CXR Database (version 2.0. 0). PhysioNet* 2019.
6. Goldberger, A. L., Amaral, L. A., Glass, L., Hausdorff, J. M., Ivanov, P. C., Mark, R. G., Mietus, J. E., Moody, G. B., Peng, C.-K. & Stanley, H. E. PhysioBank, PhysioToolkit, and PhysioNet: components of a new research resource for complex physiologic signals. *circulation* **101,** e215–e220 (2000).
7. Irvin, J., Rajpurkar, P., Ko, M., Yu, Y., Ciurea-Ilcus, S., Chute, C., Marklund, H., Haghgoo, B., Ball, R., Shpanskaya, K., *et al. Chexpert: A large chest radiograph dataset with uncertainty labels and expert comparison* in *Proceedings of the AAAI conference on artificial intelligence* **33** (2019), 590–597.
8. Wang, X., Peng, Y., Lu, L., Lu, Z., Bagheri, M. & Summers, R. M. *Chestx-ray8: Hospital-scale chest x-ray database and benchmarks on weakly-supervised classification and localization of common thorax diseases* in *Proceedings of the IEEE conference on computer vision and pattern recognition* (2017), 2097–2106.
9. Yang, L., Xu, S., Sellergren, A., Kohlberger, T., Zhou, Y., Ktena, I., Kiraly, A., Ahmed, F., Hormozdiari, F., Jaroensri, T., *et al.* Advancing multimodal medical capabilities of Gemini. *arXiv preprint arXiv:2405.03162* (2024).
10. Johnson, A., Lungren, M., Peng, Y., Lu, Z., Mark, R., Berkowitz, S. & Horng, S. *MIMIC-CXR-JPG - chest radiographs with structured labels* version 2.0.0. Nov. 2019. https://doi.org/10.13026/8360-t248.
11. Hyland, S. L., Bannur, S., Bouzid, K., Castro, D. C., Ranjit, M., Schwaighofer, A., Pérez-García, F., Salvatelli, V., Srivastav, S., Thieme, A., *et al.* MAIRA-1: A specialised large multimodal model for radiology report generation. *arXiv preprint arXiv:2311.13668* (2023).
12. Majkowska, A., Mittal, S., Steiner, D. F., Reicher, J. J., McKinney, S. M., Duggan, G. E., Eswaran, K., Cameron Chen, P.-H., Liu, Y., Kalidindi, S. R., *et al.* Chest radiograph interpretation with deep learning models: assessment with radiologist-adjudicated reference standards and population-adjusted evaluation. *Radiology* **294,** 421–431 (2020).
13. Liu, Y., Jain, A., Eng, C., Way, D. H., Lee, K., Bui, P., Kanada, K., de Oliveira Marinho, G., Gallegos, J., Gabriele, S., *et al.* A deep learning system for differential diagnosis of skin diseases. *Nature medicine* **26,** 900–908 (2020).
14. Jaroensri, R., Wulczyn, E., Hegde, N., Brown, T., Flament-Auvigne, I., Tan, F., Cai, Y., Nagpal, K., Rakha, E. A., Dabbs, D. J., *et al.* Deep learning models for histologic grading of breast cancer and association with disease prognosis. *NPJ breast cancer* **8,** 113 (2022).
15. Nagpal, K., Foote, D., Liu, Y., Chen, P.-H. C., Wulczyn, E., Tan, F., Olson, N., Smith, J. L., Mohtashamian, A., Wren, J. H., *et al.* Development and validation of a deep learning algorithm for improving Gleason scoring of prostate cancer. *NPJ digital medicine* **2,** 48 (2019).
16. Nagpal, K., Foote, D., Tan, F., Liu, Y., Chen, P.-H. C., Steiner, D. F., Manoj, N., Olson, N., Smith, J. L., Mohtashamian, A., *et al.* Development and validation of a deep learning algorithm for Gleason grading of prostate cancer from biopsy specimens. *JAMA oncology* **6,** 1372–1380 (2020).
17. Sadhwani, A., Chang, H.-W., Behrooz, A., Brown, T., Auvigne-Flament, I., Patel, H., Findlater, R., Velez, V., Tan, F., Tekiela, K., *et al.* Comparative analysis of machine learning approaches to classify tumor mutation burden in lung adenocarcinoma using histopathology images. *Scientific reports* **11,** 16605 (2021).
18. Cuadros, J. & Bresnick, G. EyePACS: an adaptable telemedicine system for diabetic retinopathy screening. *Journal of diabetes science and technology* **3,** 509–516 (2009).

19. Jain, S., Agrawal, A., Saporta, A., Truong, S. Q., Duong, D. N., Bui, T., Chambon, P., Zhang, Y., Lungren, M. P., Ng, A. Y., *et al.* Radgraph: Extracting clinical entities and relations from radiology reports. *arXiv preprint arXiv:2106.14463* (2021).

20. Tanno, R., Barrett, D., Sellergren, A., Ghaisas, S., Dathathri, S., See, A., Welbl, J., Singhal, K., Azizi, S., Tu, T., *et al.* Consensus, dissensus and synergy between clinicians and specialist foundation models in radiology report generation. *arXiv preprint arXiv:2311.18260* (2024).

21. Schmidgall, S., Ziaei, R., Harris, C., Reis, E., Jopling, J. & Moor, M. AgentClinic: a multimodal agent benchmark to evaluate AI in simulated clinical environments. *arXiv preprint arXiv:2405.07960* (2024).

22. Jin, D., Pan, E., Oufattole, N., Weng, W.-H., Fang, H. & Szolovits, P. What disease does this patient have? A large-scale open domain question answering dataset from medical exams. *Applied Sciences* **11,** 6421 (2021).

23. Johnson, A. E., Bulgarelli, L., Shen, L., Gayles, A., Shammout, A., Horng, S., Pollard, T. J., Hao, S., Moody, B., Gow, B., *et al.* MIMIC-IV, a freely accessible electronic health record dataset. *Scientific data* **10,** 1 (2023).

24. Xu, S., Yang, L., Kelly, C., Sieniek, M., Kohlberger, T., Ma, M., Weng, W.-H., Kiraly, A., Kazemzadeh, S., Melamed, Z., *et al.* ELIXR: Towards a general purpose X-ray artificial intelligence system through alignment of large language models and radiology vision encoders. *arXiv preprint arXiv:2308.01317* (2023).

25. Gatopoulos, I., Känzig, N., Moser, R. & Otálora, S. *eva: Evaluation framework for pathology foundation models* in *Medical Imaging with Deep Learning* (2024). https://openreview.net/forum?id=FNBQOPj18N.

26. Aresta, G., Araújo, T., Kwok, S., Chennamsetty, S. S., Safwan, M., Alex, V., Marami, B., Prastawa, M., Chan, M., Donovan, M., *et al.* BACH: Grand challenge on breast cancer histology images. *Medical Image Analysis* **56,** 122–139. https://doi.org/10.1016/j.media.2019.05.010 (2019).

27. Spanhol, F. A., Oliveira, L. S., Petitjean, C. & Heutte, L. A Dataset for Breast Cancer Histopathological Image Classification. *IEEE Transactions on Biomedical Engineering* **63,** 1455–1462 (2016).

28. Veeling, B. S., Linmans, J., Winkens, J., Cohen, T. & Welling, M. *Rotation Equivariant CNNs for Digital Pathology* 2018. arXiv: 1806.03962 [cs.CV]. https://arxiv.org/abs/1806.03962.

29. Kather, J. N., Halama, N. & Marx, A. *100,000 histological images of human colorectal cancer and healthy tissue* version v0.1. [Data set]. 2018. https://doi.org/10.5281/zenodo.1214456.

30. Defazio, A., Bach, F. & Lacoste-Julien, S. SAGA: A fast incremental gradient method with support for non-strongly convex composite objectives. *Advances in neural information processing systems* **27** (2014).

31. Kiraly, A. P., Baur, S., Philbrick, K., Mahvar, F., Yatziv, L., Chen, T., Sterling, B., George, N., Jamil, F., Tang, J., *et al.* Health AI Developer Foundations. *arXiv preprint arXiv:2411.15128* (2024).

32. Bejnordi, B. E., Veta, M., Van Diest, P. J., Van Ginneken, B., Karssemeijer, N., Litjens, G., Van Der Laak, J. A., Hermsen, M., Manson, Q. F., Balkenhol, M., *et al.* Diagnostic assessment of deep learning algorithms for detection of lymph node metastases in women with breast cancer. *Jama* **318,** 2199–2210 (2017).

33. Lai, J., Ahmed, F., Vijay, S., Jaroensri, T., Loo, J., Vyawahare, S., Agarwal, S., Jamil, F., Matias, Y., Corrado, G. S., *et al.* Domain-specific optimization and diverse evaluation of self-supervised models for histopathology. *arXiv preprint arXiv:2310.13259* (2023).

34. Wu, J., Agu, N., Lourentzou, I., Sharma, A., Paguio, J., Yao, J. S., Dee, E. C., Mitchell, W., Kashyap, S., Giovannini, A., *et al.* Chest imagenome dataset. *Physio Net* (2021).

35. Pampari, A., Raghavan, P., Liang, J. & Peng, J. *emrQA: A Large Corpus for Question Answering on Electronic Medical Records* in *Proceedings of the 2018 Conference on Empirical Methods in Natural Language Processing* (Association for Computational Linguistics, Oct. 2018), 2357–2368. https://aclanthology.org/D18-1258/.

36. Raghavan, P., Liang, J. J., Mahajan, D., Chandra, R. & Szolovits, P. *emrKBQA: A Clinical Knowledge-Base Question Answering Dataset* in *Proceedings of the 20th Workshop on Biomedical Language Processing* (Association for Computational Linguistics, June 2021), 64–73. https://aclanthology.org/2021.bionlp-1.7/.

37. Lee, G., Kweon, S., Bae, S. & Choi, E. *Overview of the EHRSQL 2024 Shared Task on Reliable Text-to-SQL Modeling on Electronic Health Records* in *Proceedings of the 6th Clinical Natural Language Processing Workshop* (Association for Computational Linguistics, June 2024), 644–654. https://aclanthology.org/2024.clinicalnlp-1.62/.

38. Kweon, S., Kim, J., Kwak, H., Cha, D., Yoon, H., Kim, K. H., Yang, J., Won, S. & Choi, E. *EHRNoteQA: An LLM Benchmark for Real-World Clinical Practice Using Discharge Summaries* PhysioNet, version 1.0.1. June 2024. https://doi.org/10.13026/acga-ht95.

39. Fleming, S. L., Lozano, A., Haberkorn, W. J., Jindal, J. A., Reis, E., Thapa, R., Blankemeier, L., Genkins, J. Z., Steinberg, E., Nayak, A., *et al.* MedAlign: A Clinician-Generated Dataset for Instruction Following with Electronic Medical Records. *Proceedings of the AAAI Conference on Artificial Intelligence* **38,** 22021–22030. https://ojs.aaai.org/index.php/AAAI/article/view/30205 (Mar. 2024).

40. Walonoski, J., Kramer, M., Nichols, J., Quina, A., Moesel, C., Hall, D., Duffett, C., Dube, K., Gallagher, T. & McLachlan, S. Synthea: An approach, method, and software mechanism for generating synthetic patients and the synthetic electronic health care record. *Journal of the American Medical Informatics Association* **25,** 230–238 (2018).

41. Yang, Z., Qi, P., Zhang, S., Bengio, Y., Cohen, W., Salakhutdinov, R. & Manning, C. D. *HotpotQA: A Dataset for Diverse, Explainable Multi-hop Question Answering* in *Proceedings of the 2018 Conference on Empirical Methods in Natural Language Processing* (Association for Computational Linguistics, Brussels, Belgium, Oct. 2018), 2369–2380. https://aclanthology.org/D18-1259/.

42. Evbayekha, E., Idowu, A. B. & LaRue, S. Sacubitril/Valsartan vs ACE Inhibitors or ARBs: A Systematic Review and Meta-Analysis of Randomized Trials. *JACC: Advances* **4,** 101598 (2025).
